# Supplementary material for: AKT mutant allele-specific activation dictates pharmacologic sensitivities
Source: Nat Commun. 2022 Apr 19;13:2111. doi: 10.1038/s41467-022-29638-1 (PMC9018718; doi:10.1038/s41467-022-29638-1)
Supplement: Supplementary file 1 — Supplementary information along with the clinical protocol [file 41467_2022_29638_MOESM1_ESM.pdf]

AKT mutant allele-specific activation dictates pharmacologic sensitivities

Tripti Shrestha Bhattarai[1,2], Tambudzai Shamu[1,2], Alexander N. Gorelick[1,2], Matthew T. Chang[1,2,6], Debyani Chakravarty[3], Elena I. Gavrila[1,2], Mark T.A. Donoghue[3], JianJong Gao[3], Swati Patel[1], Sizhi Paul Gao[1], Margaret H. Reynolds[4], Sarah M. Phillips[3], Tara Soumerai[4,7], Wassim Abida[4], David M. Hyman[4,5], Alison M. Schram[4], David B. Solit[1,3,4,5], Lillian M. Smyth[4,8], Barry S. Taylor[1,2,3,5,8]

Supplementary Materials

Supplementary Tables

Supplementary Table 1: Data-driven identification of candidate driver mutations in *AKT1*, *AKT2*, and *AKT3*. The mutant alleles identified by population-scale computational analysis of 41,075 sequenced primary and advanced cancers.

Gene	Residue	Mutation Type	Number of mutant cases	Prediction
AKT1	E17	Missense	338	Hotspot, 3D, OncoKB
AKT1	L52	Missense	15	Hotspot, OncoKB
AKT1	D323	Missense	12	Hotspot, 3D, OncoKB
AKT1	E40	Missense	8	Hotspot, OncoKB
AKT1	Q79	Missense	8	Hotspot, OncoKB
AKT1	W80	Missense	7	Hotspot, OncoKB
AKT1	T65-C77	Indel	6	Hotspot, Paralogy
AKT1	E322	Missense	4	3D
AKT1	R25	Missense	3	3D
AKT1	R15	Missense	2	3D
AKT1	R23	Missense	2	3D
AKT1	G311	Missense	1	OncoKB
AKT1	V320	Missense	1	3D
AKT1	L321	Missense	1	3D
AKT1	C77	Missense	0	OncoKB
AKT1	K179	Missense	0	OncoKB
AKT2	D324	Missense	6	Hotspot
AKT2	E17	Missense	5	OncoKB, Paralogy
AKT2	C60-I84	Indel	5	Hotspot, Paralogy
AKT2	R368	Missense	4	OncoKB
AKT2	L52	Missense	3	Paralogy
AKT2	E323	Missense	3	Paralogy
AKT2	R25	Missense	2	Paralogy
AKT2	E40	Missense	1	Paralogy
AKT2	Q79	Missense	1	Paralogy
AKT2	W80	Missense	1	Paralogy
AKT2	V321	Missense	0	Paralogy
AKT2	L322	Missense	0	Paralogy

AKT3	E17	Missense	13	Hotspot, OncoKB, Paralogy
AKT3	Q78	Missense	5	Paralogy
AKT3	R15	Missense	3	Paralogy
AKT3	E40	Missense	2	Paralogy
AKT3	L51	Missense	2	Paralogy
AKT3	R25	Missense	1	Paralogy
AKT3	E319	Missense	1	Paralogy
AKT3	D320	Missense	1	Paralogy
AKT3	W79	Missense	0	Paralogy
AKT3	V317	Missense	0	Paralogy
AKT3	L318	Missense	0	Paralogy

Supplementary Table 2: IC_{50} data for all tested AKT inhibitors (ATP-competitive inhibitor capivasertib and the allosteric inhibitors ARQ092 and MK2206) in cells expressing each of the indicated *AKT1* and *AKT2* mutations.

		IC50 (uM)			
**Gene**	**Mutant**	**capivasertib**	**ARQ092**	**MK2206**	**Status[a]**
AKT1	WT	40.1	5.8	9.5	Wildtype
AKT1	R15Q	125.6	6.1	6.5	NA
AKT1	E17K	7.2	3.5	5.7	AM
AKT1	W22R	61.2	11.8	19.2	NA
AKT1	E40K	9.5	3.1	2.5	AM
AKT1	D44N	50.8	9.0	6.7	NA
AKT1	R48H	55.4	6.5	7.5	NA
AKT1	L52R	15.5	6.3	6.8	AM
AKT1	F55Y	11.5	5.0	7.7	AM
AKT1	T65-I75dup	4.8	13.8	52.5	AI
AKT1	E66-Q79dup	4.1	33.0	78.3	AI
AKT1	P68-C77dup	2.7	41.9	88.3	AI
AKT1	Q79K	5.8	4.7	3.3	AM
AKT1	W80R	8.8	6.6	61.8	AM
AKT1	E267G	84.7	8.5	10.9	NA
AKT1	D323G	9.1	4.0	3.9	AM
AKT1	E341K	32.9	9.3	9.9	NA
AKT1	R370C	61.3	6.4	5.0	NA
AKT1	E464K	60.5	7.3	4.9	NA
AKT2	WT	23.9	3.7	-	Wildtype
AKT2	E17K	8.1	4.4	-	AM
AKT2	60_75dup	6.4	4.1	-	AI
AKT2	67_78dup	6.6	5.3	-	AI
AKT2	68_80dup	5.3	24.2	-	AI
AKT2	76_84dup	4.9	22.3	-	AI
AKT2	78_79ins	4.4	16.9	-	AI

[a] AM, activating missense; AI, activating indel; NA, not activating

Supplementary Table 3: Baseline characteristics of enrolled patients. Shown here are the baseline characteristics of the patients enrolled in the investigator-initiated trial of capivasertib treatment in *AKT1-3* mutant solid cancers.

**Characteristic**	**Value**
Age, years	
Median (range)	65 (33-72)
Sex, n (%)	
Female	7 (58)
Male	5 (36)
ECOG Performance status, n (%)	
0	1 (8)
1	11 (92)
No. of previous systemic chemotherapies, n (%)	
0-1	3 (25)
2	1 (8)
3-4	2 (17)
≥5	6 (50)
Tumor type, n (%)	
Prostate	3 (25)
Breast	2 (17)
ER+	1 (8)
TNBC	1 (8)
Soft-tissue sarcoma	2 (17)
Leiomyosarcoma	1 (8)
Anal	1 (8)
Endometrial	1 (8)
Cervical	1 (8)
Tonsillar	1 (8)
CNS metastases, n (%)	
No	12 (100)
Qualifying mutation, n (%)	
AKT1 E17K	6 (50)
AKT2/3 E17K	2 (17)
AKT1/2 non-E17K	4 (33)

Supplementary Table. 4: Treatment-emergent adverse events (AEs). Those AEs occurring in >15% of patients overall and AEs of grade ≥3 severity regardless of causality

	Patients, n (%)				
**Adverse event [a]**	**Grade 1**	**Grade 2**	**Grade 3**	**Grade 4**	**Grade 5**
Diarrhea	3 (25)	3 (25)	0	0	0
Nausea	3 (25)	2 (17)	0	0	0
Anemia	0	0	4 (33)	0	0
Pain	0	1 (8)	3 (25)	0	0
Anorexia	3 (25)	1 (8)	0	0	0
Fatigue	1 (8)	1 (8)	1 (8)	0	0
Vomiting	3 (25)	0	0	0	0
Rash maculopapular	3 (25)	0	0	0	0
Acute kidney injury	0	0	1 (8)	0	0
Dyspnea	0	2 (17)	0	0	0
Abdominal pain	1 (8)	1 (8)	0	0	0
Productive cough	2 (17)	0	0	0	0
Hyperglycemia	0	0	0	1 (8)	0
Hypokalemia	0	0	1 (8)	0	0
Bacteremia	0	0	1 (8)	0	0

[a] The highest grade of a given toxicity is reported per patient. Laboratory toxicity is included if considered clinically significant.

Supplementary Figures

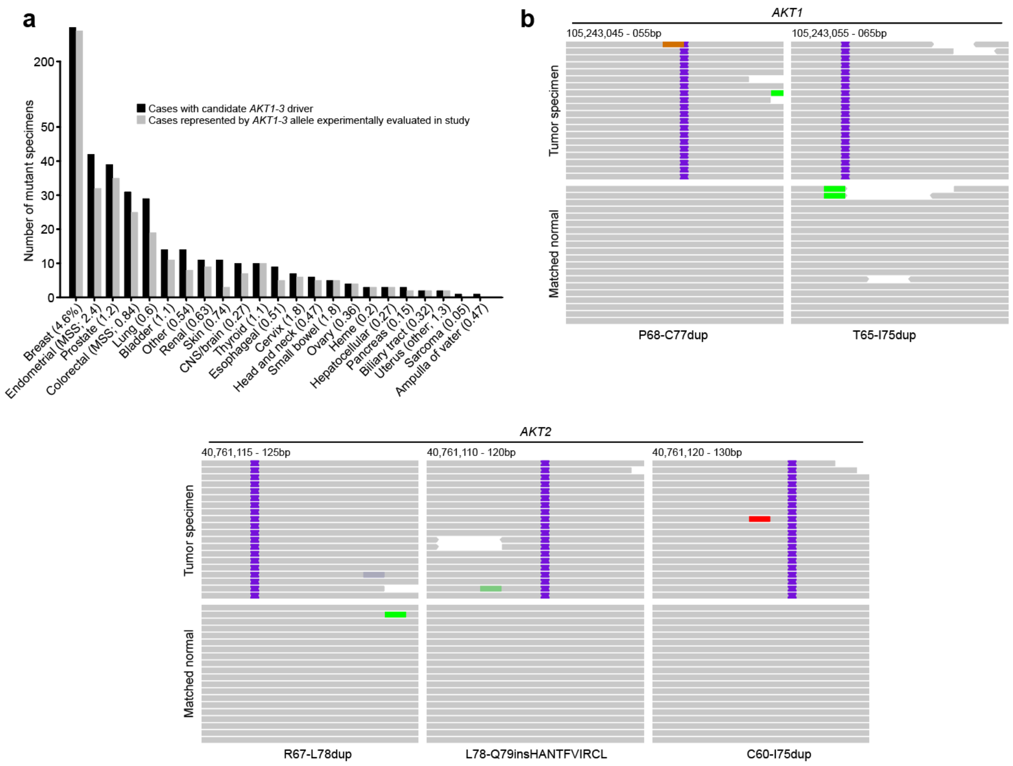

Supplementary Fig. 1: Representation of candidate driver *AKT* mutants identified and tested. a) The overall representation of disease types among patients with candidate *AKT1-3* driver mutations identified here and the subset encompassed by the set of mutant alleles we functionally characterized in the current study (the total incidence of *AKT1-3* mutations by tumor type are included in parentheses from Figure 1 for reference). **b)** The sequencing reads spanning the site of representative *AKT1* (top right) and *AKT2* (bottom) indels in affected tumors and matched normal controls (as labeled).

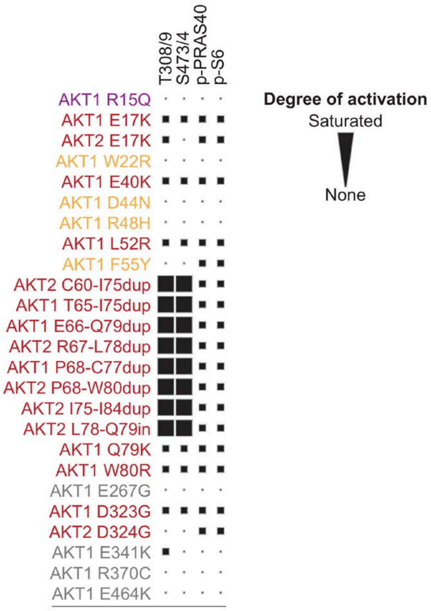

Supplementary Fig. 2: AKT mutant activation. Summary of the activation of p-AKT and downstream targets by myriad missense and in-frame mutations in AKT1 and AKT2 assessed here.

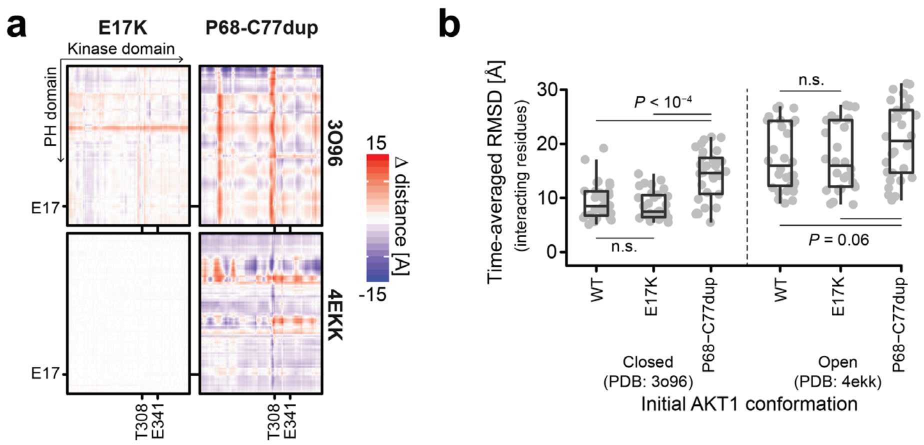

Supplementary Fig. 3: Structural impacts in open conformation. a) MD simulations as described in the main text were also performed using a structure of AKT1 that was ATP analog-bound and therefore in open conformation (4EKK). This analysis indicated that the P68-C77dup had a more profound structural effect on AKT1 in closed conformation (3O96; allosteric inhibitor-bound) as compared to ATP-bound AKT1 in open conformation. **b)** MD simulations of AKT1 WT, E17K, and P68-C77dup alleles for 100ns using as templates both PDB structure 3O96 (left; an allosteric inhibitor-bound AKT1, representing its closed conformation) and 4EKK (right; AMP-PNP bound, representing an open, ATP-bound conformation) from which the root-mean-squared-distances (RMSD) between the time-averaged positions for each 37 pairs of strongly interacting residues producing hydrophobic interactions between the PH and kinase domains was determined (see Methods). In closed conformation, P68-C77dup mutation resulted in a significant increase in the distances between interacting residues (median RMSD=14.6Å) compared to WT (median RMSD=8.5Å) (P = 7x10^{-5}, two-sided unpaired Wilcoxon rank sum test). N.S., not significant. Conversely, when simulated in an open conformation where the interacting residues in each allele had large initial distances (median RMSD ≥ 16Å), P68-C77dup had a weaker effect on the distance between interacting residues (median RMSD=20.6Å compared to 16Å in WT, P=0.06), and as before E17K did not significantly affect the interacting residues. The center of the boxes represents the median value, and the upper/lower box bounds are the 25th-75th percentiles. The upper and lower whiskers show the most extreme values within 1.5 times the inter-quartile range (IQR) from the upper and lower box bounds, respectively.

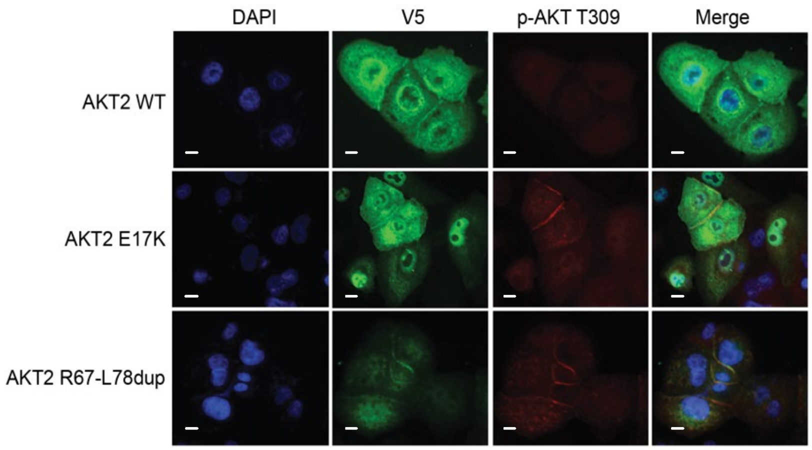

Supplementary Fig. 4: Mutant AKT2 localization. Immunofluorescence (IF) microscopy of MCF10a cells stably expressing AKT2 WT, E17K, or R67-L78dup in assay media for four hours. WT AKT2 was primarily cytoplasmic in localization while E17K-mutant cells were partially membrane-localized and the R67-L78dup-mutant cells were predominantly localized to the membrane, consistent with findings for similar mutations in *AKT1*. Images are representative of two independent experiments. Scale bar: 40um.

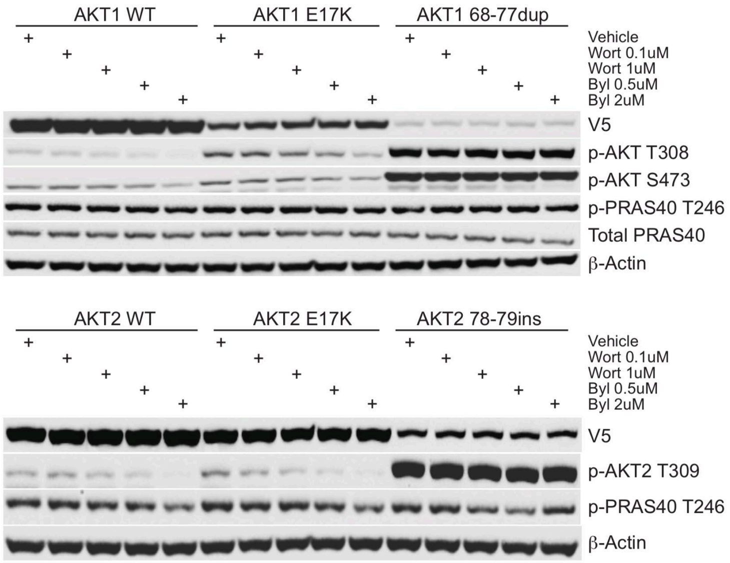

Supplementary Fig. 5: The role of PIP3 formation in mutant allele-specific binding affinity. In MCF10a cells stably expressing AKT1 WT, AKT1 E17K, and AKT1 P68-C77dup (top) or AKT2 WT, AKT2 E17K, and AKT2 78-79ins(HANTFVIRCL) (bottom), we assessed the role of PIP3 in binding affinity differences among the structurally distinct mutants. PIP3 formation was inhibited in cells treated with either Wortmannin (non-specific covalent inhibitor of the PI3Ks) or BYL-719 (PIK3CA inhibitor). Increasing concentrations of both agents led to diminished p-Akt in both WT and E17K cells, suggesting their dependence on PIP3 formation for pathway activation. By contrast, activation by the indel mutants were independent of PIP3 conversion, as indicated by their p-AKT levels. Results were obtained from two independent experiments. Source data are provided as a Source Data file.

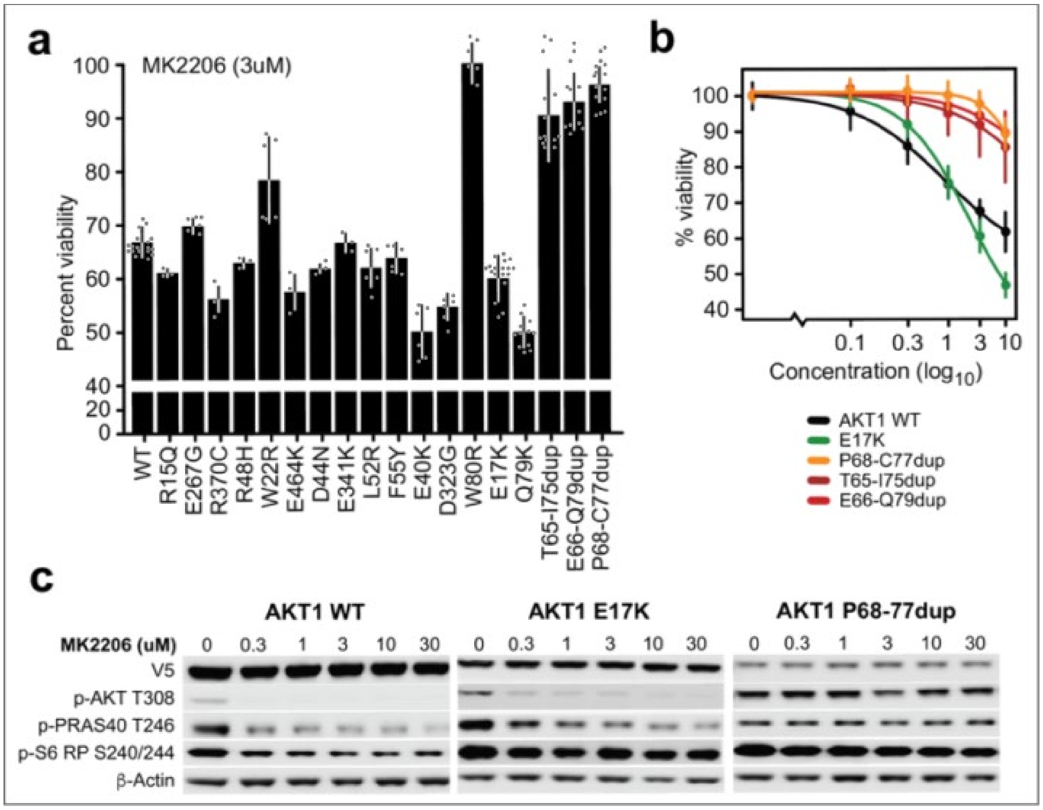

Supplementary Fig. 6: Sensitivity of *AKT1* mutants to MK2206. a) The viability of MCF10a cells expressing either WT AKT1 or the indicated mutants treated with the allosteric AKT inhibitor MK2206 (3uM) for 72 hours. Activating *AKT1* indel mutations are insensitive to MK2206 treatment (as is missense W80R). **b)** Cells expressing WT, E17K, or three indel mutants (as in panel a) were treated with the indicated concentrations of MK2206. Cell viability was assessed 72 hours post-treatment. The values in Supp. Figures 6a and 6b have been derived from at least three independent experiments with triplicates in each experiment. Error bars represent standard deviations from the means. **c)** MCF10a cells expressing the indicated mutants were treated with multiple concentrations of MK2206, whole cell lysates were harvested four hours post-treatment, and pathway inhibition was assessed by western blot. The immunoblot shown is representative of two independent experiments. Source data are provided as a Source Data file.

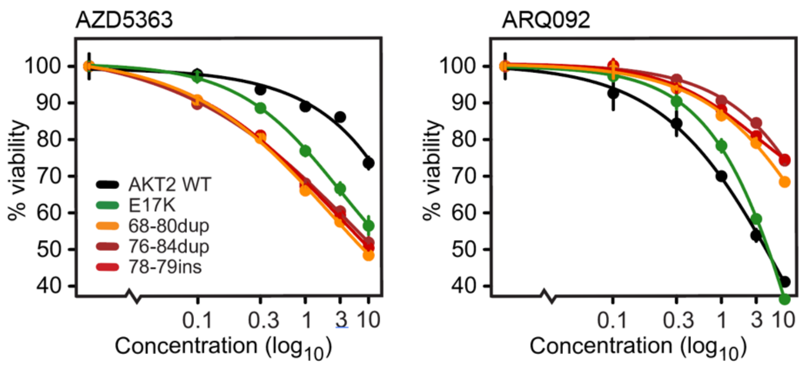

Supplementary Fig. 7: *AKT2* mutant sensitivity to AKT inhibition. The viability of MCF10a cells stably expressing various *AKT2* mutants indicate that while the E17K hotspot is sensitive to multiple AKT inhibitors independent of their mechanisms of action, the in-frame indel mutants have the greatest sensitivity to ATP-competitive inhibitor capivasertib (AZD5363, left) and were refractory to allosteric inhibitor therapy with ARQ092 (right), similar to activating *AKT1* mutations. Error bars are standard deviations from the mean. Source data are provided as a Source Data file.

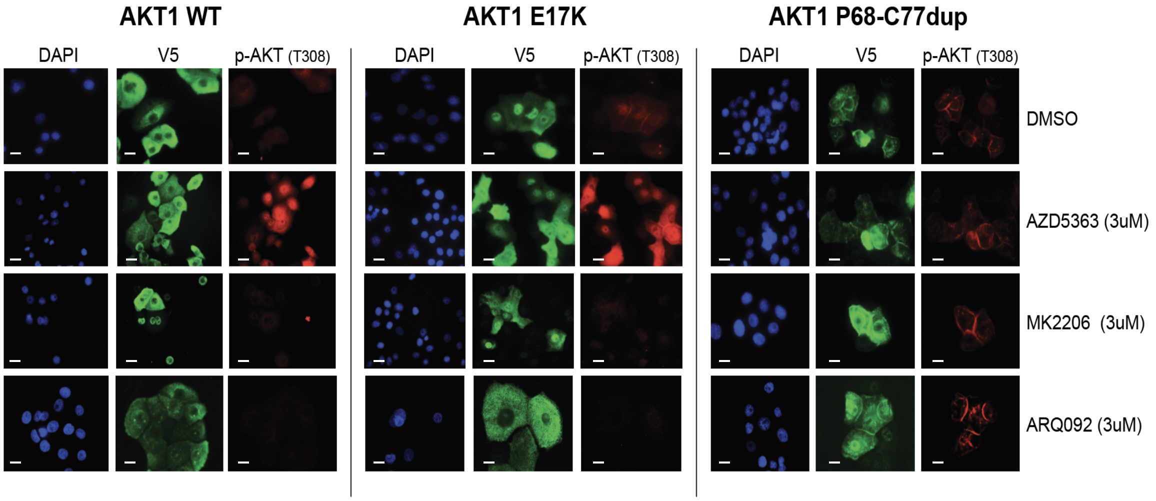

Supplementary Fig. 8: *AKT1* indels are resistant to allosteric *AKT* inhibitors. MCF10a cells stably expressing WT AKT1, E17K or P68-C77dup mutants seeded in chamber slides were treated with either the ATP-competitive AKT inhibitor capivasertib (AZD5363), or allosteric AKT inhibitors MK2206 and ARQ092 (3uM) in assay media. Four hours post treatment, cells were fixed and tagged with antibodies and fluorescent probes for V5 (*AKT* expression) and p-AKT (T-308) signals, and analyzed by immunofluorescence (IF) microscopy. Images are representative of two independent experiments. Scale bar: 40um.

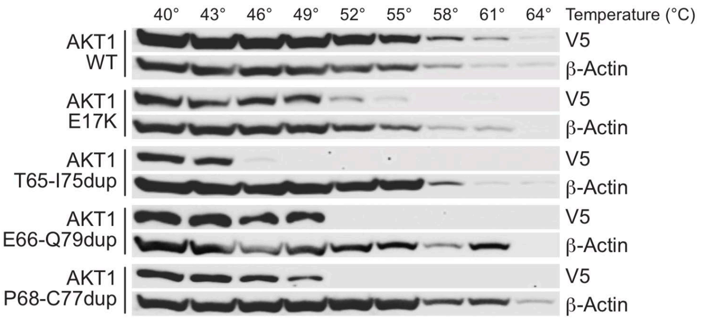

Supplementary Fig. 9: Thermal stability analysis of mutant AKT1. Thermal shift assays evaluating the structural consequences of *AKT1* missense and indel mutations. Cells expressing WT AKT1 (in closed conformation) remained stable through a higher temperature range (melting temperature T_m) than did AKT1 E17K, which appears modestly less stable. AKT1 indels, by contrast, readily denature at lower temperatures, likely due to their open conformation and structural destabilization resulting from the loss of stabilizing inter-domain interactions as suggested by our MD simulation studies. The immunoblot shown is representative of two independent experiments. Source data are provided as a Source Data file.

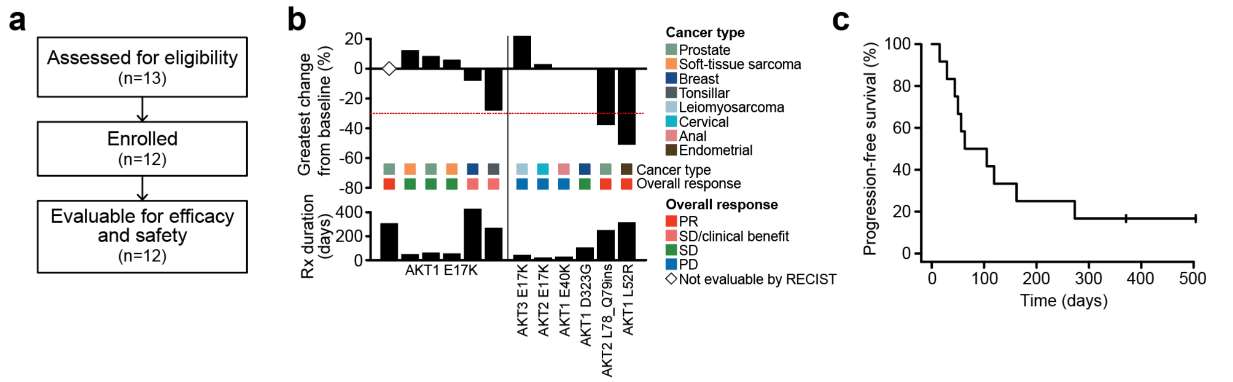

Supplementary Fig. 10: AKT inhibition in AKT1-3 mutant solid cancers. a) Consort diagram representing the clinical trial reported here. **b)** The clinical response of patients with either *AKT1* E17K- (left) or other *AKT1-3* driver-mutant tumors (right) solid cancers to capivasertib therapy is shown. The objective response rate was 25% (n = 3 of 12) and the clinical benefit rate was 42% (n = 5 of 12). **c)** Progression-free survival of all patients on capivasertib therapy (median progression-free survival 84 days; 95% CI 50-not reached).

References

1. Bellacosa, A., Testa, J. R., Staal, S. P. & Tsichlis, P. N. A retroviral oncogene, akt, encoding a serine-threonine kinase containing an SH2-like region. *Science* **254,** 274–277 (1991).
2. Scheid, M. P. & Woodgett, J. R. PKB/AKT: functional insights from genetic models. *Nat. Rev. Mol. Cell Biol.* **2,** 760–768 (2001).
3. Manning, B. D. & Toker, A. AKT/PKB signaling: navigating the network. *Cell* **169,** 381–405 (2017).
4. Carpten, J. D. *et al.* A transforming mutation in the pleckstrin homology domain of AKT1 in cancer. *Nature* **448,** 439–444 (2007).
5. Mundi, P. S., Sachdev, J., McCourt, C. & Kalinsky, K. AKT in cancer: new molecular insights and advances in drug development. *Br. J. Clin. Pharmacol.* **82,** 943–956 (2016).
6. Chang, M. T. *et al.* Identifying recurrent mutations in cancer reveals widespread lineage diversity and mutational specificity. *Nat. Biotechnol.* **34,** 155–163 (2016).
7. Parikh, C. *et al.* Disruption of PH-kinase domain interactions leads to oncogenic activation of AKT in human cancers. *Proc. Natl. Acad. Sci. USA* **109,** 19368–19373 (2012).
8. Dutt, A. *et al.* Somatic mutations are present in all members of the AKT family in endometrial carcinoma. *Br. J. Cancer* **101,** 1218–9; author reply 1220 (2009).
9. Hyman, D. M. *et al.* AKT inhibition in solid tumors with AKT1 mutations. *J. Clin. Oncol.* **35,** 2251–2259 (2017).
10. Davies, B. R. *et al.* Tumors with AKT1E17K Mutations Are Rational Targets for Single Agent or Combination Therapy with AKT Inhibitors. *Mol. Cancer Ther.* **14,** 2441–2451 (2015).
11. Chang, M. T. *et al.* Accelerating discovery of functional mutant alleles in cancer. *Cancer Discov.* **8,** 174–183 (2018).
12. Zehir, A. *et al.* Mutational landscape of metastatic cancer revealed from prospective clinical sequencing of 10,000 patients. *Nat. Med.* **23,** 703–713 (2017).
13. Gao, J. *et al.* 3D clusters of somatic mutations in cancer reveal numerous rare mutations as functional targets. *Genome Med.* **9,** 4 (2017).
14. Chakravarty, D. *et al.* Oncokb: A precision oncology knowledge base. *JCO Precis. Oncol.* **2017,** (2017).
15. Zhang, Y. *et al.* A Pan-Cancer Proteogenomic Atlas of PI3K/AKT/mTOR Pathway Alterations. *Cancer Cell* **31,** 820–832.e3 (2017).
16. Cancer Genome Atlas Network. Comprehensive molecular portraits of human breast tumours. *Nature* **490,** 61–70 (2012).
17. Cancer Genome Atlas Research Network *et al.* The Cancer Genome Atlas Pan-Cancer analysis project. *Nat. Genet.* **45,** 1113–1120 (2013).
18. Kim, E. *et al.* Systematic functional interrogation of rare cancer variants identifies oncogenic alleles. *Cancer Discov.* **6,** 714–726 (2016).
19. Thomas, C. C., Deak, M., Alessi, D. R. & van Aalten, D. M. F. High-resolution structure of the pleckstrin homology domain of protein kinase b/akt bound to phosphatidylinositol (3,4,5)-trisphosphate. *Curr. Biol.* **12,** 1256–1262 (2002).
20. Calleja, V., Laguerre, M., Parker, P. J. & Larijani, B. Role of a novel PH-kinase domain interface in PKB/Akt regulation: structural mechanism for allosteric inhibition. *PLoS Biol.* **7,** e17 (2009).
21. Calleja, V. *et al.* Intramolecular and intermolecular interactions of protein kinase B define its activation in vivo. *PLoS Biol.* **5,** e95 (2007).
22. Wu, W.-I. *et al.* Crystal structure of human AKT1 with an allosteric inhibitor reveals a new mode of kinase inhibition. *PLoS One* **5,** e12913 (2010).

23. Lin, K. *et al.* An ATP-site on-off switch that restricts phosphatase accessibility of Akt. *Sci. Signal.* **5,** ra37 (2012).
24. Kohn, A. D., Takeuchi, F. & Roth, R. A. Akt, a pleckstrin homology domain containing kinase, is activated primarily by phosphorylation. *J. Biol. Chem.* **271,** 21920–21926 (1996).
25. Lučić, I. *et al.* Conformational sampling of membranes by Akt controls its activation and inactivation. *Proc. Natl. Acad. Sci. USA* **115,** E3940–E3949 (2018).
26. Okuzumi, T. *et al.* Inhibitor hijacking of Akt activation. *Nat. Chem. Biol.* **5,** 484–493 (2009).
27. Vivanco, I. *et al.* A kinase-independent function of AKT promotes cancer cell survival. *Elife* **3,** (2014).
28. Green, C. J. *et al.* Use of Akt inhibitor and a drug-resistant mutant validates a critical role for protein kinase B/Akt in the insulin-dependent regulation of glucose and system A amino acid uptake. *J. Biol. Chem.* **283,** 27653–27667 (2008).
29. Yu, Y. *et al.* Targeting AKT1-E17K and the PI3K/AKT Pathway with an Allosteric AKT Inhibitor, ARQ 092. *PLoS One* **10,** e0140479 (2015).
30. Smyth, L. M. *et al.* Capivasertib, an AKT Kinase Inhibitor, as Monotherapy or in Combination with Fulvestrant in Patients with AKT1E17K-Mutant, ER-Positive Metastatic Breast Cancer. *Clin. Cancer Res.* **26,** 3947–3957 (2020).
31. Harrington, L. S. *et al.* The TSC1-2 tumor suppressor controls insulin-PI3K signaling via regulation of IRS proteins. *J. Cell Biol.* **166,** 213–223 (2004).
32. O’Reilly, K. E. *et al.* mTOR inhibition induces upstream receptor tyrosine kinase signaling and activates Akt. *Cancer Res.* **66,** 1500–1508 (2006).
33. You, H.-L. *et al.* Association of IRS2 overexpression with disease progression in intrahepatic cholangiocarcinoma. *Oncol. Lett.* **16,** 5505–5511 (2018).
34. Dearth, R. K., Cui, X., Kim, H.-J., Hadsell, D. L. & Lee, A. V. Oncogenic transformation by the signaling adaptor proteins insulin receptor substrate (IRS)-1 and IRS-2. *Cell Cycle* **6,** 705–713 (2007).
35. Yao, Z. *et al.* Tumours with class 3 BRAF mutants are sensitive to the inhibition of activated RAS. *Nature* **548,** 234–238 (2017).
36. Gao, Y. *et al.* Allele-Specific Mechanisms of Activation of MEK1 Mutants Determine Their Properties. *Cancer Discov.* **8,** 648–661 (2018).
37. Ellrott, K. *et al.* Scalable open science approach for mutation calling of tumor exomes using multiple genomic pipelines. *Cell Syst.* **6,** 271–281.e7 (2018).
38. Cheng, D. T. *et al.* Memorial Sloan Kettering-Integrated Mutation Profiling of Actionable Cancer Targets (MSK-IMPACT): A Hybridization Capture-Based Next-Generation Sequencing Clinical Assay for Solid Tumor Molecular Oncology. *J Mol Diagn* **17,** 251–264 (2015).
39. Chu, N. *et al.* Akt kinase activation mechanisms revealed using protein semisynthesis. *Cell* **174,** 897–907.e14 (2018).
40. Roy, A., Kucukural, A. & Zhang, Y. I-TASSER: a unified platform for automated protein structure and function prediction. *Nat. Protoc.* **5,** 725–738 (2010).
41. Yang, J. *et al.* The I-TASSER Suite: protein structure and function prediction. *Nat. Methods* **12,** 7–8 (2015).
42. Xu, J. & Zhang, Y. How significant is a protein structure similarity with TM-score = 0.5? *Bioinformatics* **26,** 889–895 (2010).
43. Pettersen, E. F. *et al.* UCSF Chimera—a visualization system for exploratory research and analysis. *J. Comput. Chem.* **25,** 1605–1612 (2004).
44. Webb, B. & Sali, A. Comparative protein structure modeling using MODELLER. *Curr Protoc Protein Sci* **86,** 2.9.1–2.9.37 (2016).
45. Zhang, Y. & Skolnick, J. TM-align: a protein structure alignment algorithm based on the TM-score. *Nucleic Acids Res.* **33,** 2302–2309 (2005).

46. Pronk, S. *et al.* GROMACS 4.5: a high-throughput and highly parallel open source molecular simulation toolkit. *Bioinformatics* **29,** 845–854 (2013).
47. Van Der Spoel, D. *et al.* GROMACS: fast, flexible, and free. *J. Comput. Chem.* **26,** 1701–1718 (2005).
48. Abraham, M. J. *et al.* GROMACS: High performance molecular simulations through multi-level parallelism from laptops to supercomputers. *SoftwareX* **1-2,** 19–25 (2015).
49. Duan, Y. *et al.* A point-charge force field for molecular mechanics simulations of proteins based on condensed-phase quantum mechanical calculations. *J. Comput. Chem.* **24,** 1999–2012 (2003).
50. Berendsen, H. J. C., Grigera, J. R. & Straatsma, T. P. The missing term in effective pair potentials. *J. Phys. Chem.* **91,** 6269–6271 (1987).
51. Hess, B., Bekker, H., Berendsen, H. J. C. & Fraaije, J. G. E. M. LINCS: A linear constraint solver for molecular simulations. *J. Comput. Chem.* **18,** 1463–1472 (1997).
52. Miyamoto, S. & Kollman, P. A. Settle: An analytical version of the SHAKE and RATTLE algorithm for rigid water models. *J. Comput. Chem.* **13,** 952–962 (1992).
53. Páll, S. & Hess, B. A flexible algorithm for calculating pair interactions on SIMD architectures. *Comput Phys Commun* **184,** 2641–2650 (2013).
54. Essmann, U. *et al.* A smooth particle mesh Ewald method. *J. Chem. Phys.* **103,** 8577 (1995).
55. Bussi, G., Donadio, D. & Parrinello, M. Canonical sampling through velocity rescaling. *J. Chem. Phys.* **126,** 014101 (2007).
56. Parrinello, M. Polymorphic transitions in single crystals: A new molecular dynamics method. *J. Appl. Phys.* **52,** 7182 (1981).
57. Ferreira de Freitas, R. & Schapira, M. A systematic analysis of atomic protein-ligand interactions in the PDB. *Medchemcomm* **8,** 1970–1981 (2017).
58. Porollo, A. & Meller, J. Prediction-based fingerprints of protein-protein interactions. *Proteins* **66,** 630–645 (2007).
59. Eisenhauer, E. A. *et al.* New response evaluation criteria in solid tumours: revised RECIST guideline (version 1.1). *Eur. J. Cancer* **45,** 228–247 (2009).
60. Gorelick, A. Companion code for article "AKT mutant allele-specific activation dictates pharmacologic sensitivities. *Zenodo* (2021). doi:10.5281/zenodo.5111040

Methods-only References

6. Chang, M. T. *et al.* Identifying recurrent mutations in cancer reveals widespread lineage diversity and mutational specificity. *Nat. Biotechnol.* **34,** 155–163 (2016).
11. Chang, M. T. *et al.* Accelerating discovery of functional mutant alleles in cancer. *Cancer Discov.* **8,** 174–183 (2018).
12. Zehir, A. *et al.* Mutational landscape of metastatic cancer revealed from prospective clinical sequencing of 10,000 patients. *Nat. Med.* **23,** 703–713 (2017).
13. Gao, J. *et al.* 3D clusters of somatic mutations in cancer reveal numerous rare mutations as functional targets. *Genome Med.* **9,** 4 (2017).
22. Wu, W.-I. *et al.* Crystal structure of human AKT1 with an allosteric inhibitor reveals a new mode of kinase inhibition. *PLoS One* **5,** e12913 (2010).
23. Lin, K. *et al.* An ATP-site on-off switch that restricts phosphatase accessibility of Akt. *Sci. Signal.* **5,** ra37 (2012).
37. Ellrott, K. *et al.* Scalable open science approach for mutation calling of tumor exomes using multiple genomic pipelines. *Cell Syst.* **6,** 271–281.e7 (2018).
38. Cheng, D. T. *et al.* Memorial Sloan Kettering-Integrated Mutation Profiling of Actionable Cancer Targets (MSK-IMPACT): A Hybridization Capture-Based Next-Generation

Sequencing Clinical Assay for Solid Tumor Molecular Oncology. *J Mol Diagn* **17,** 251–264 (2015).
39. Chu, N. *et al.* Akt kinase activation mechanisms revealed using protein semisynthesis. *Cell* **174,** 897–907.e14 (2018).
40. Roy, A., Kucukural, A. & Zhang, Y. I-TASSER: a unified platform for automated protein structure and function prediction. *Nat. Protoc.* **5,** 725–738 (2010).
41. Yang, J. *et al.* The I-TASSER Suite: protein structure and function prediction. *Nat. Methods* **12,** 7–8 (2015).
42. Xu, J. & Zhang, Y. How significant is a protein structure similarity with TM-score = 0.5? *Bioinformatics* **26,** 889–895 (2010).
43. Pettersen, E. F. *et al.* UCSF Chimera—a visualization system for exploratory research and analysis. *J. Comput. Chem.* **25,** 1605–1612 (2004).
44. Webb, B. & Sali, A. Comparative protein structure modeling using MODELLER. *Curr Protoc Protein Sci* **86,** 2.9.1–2.9.37 (2016).
45. Zhang, Y. & Skolnick, J. TM-align: a protein structure alignment algorithm based on the TM-score. *Nucleic Acids Res.* **33,** 2302–2309 (2005).
46. Pronk, S. *et al.* GROMACS 4.5: a high-throughput and highly parallel open source molecular simulation toolkit. *Bioinformatics* **29,** 845–854 (2013).
47. Van Der Spoel, D. *et al.* GROMACS: fast, flexible, and free. *J. Comput. Chem.* **26,** 1701–1718 (2005).
48. Abraham, M. J. *et al.* GROMACS: High performance molecular simulations through multi-level parallelism from laptops to supercomputers. *SoftwareX* **1-2,** 19–25 (2015).
49. Duan, Y. *et al.* A point-charge force field for molecular mechanics simulations of proteins based on condensed-phase quantum mechanical calculations. *J. Comput. Chem.* **24,** 1999–2012 (2003).
50. Berendsen, H. J. C., Grigera, J. R. & Straatsma, T. P. The missing term in effective pair potentials. *J. Phys. Chem.* **91,** 6269–6271 (1987).
51. Hess, B., Bekker, H., Berendsen, H. J. C. & Fraaije, J. G. E. M. LINCS: A linear constraint solver for molecular simulations. *J. Comput. Chem.* **18,** 1463–1472 (1997).
52. Miyamoto, S. & Kollman, P. A. Settle: An analytical version of the SHAKE and RATTLE algorithm for rigid water models. *J. Comput. Chem.* **13,** 952–962 (1992).
53. Páll, S. & Hess, B. A flexible algorithm for calculating pair interactions on SIMD architectures. *Comput Phys Commun* **184,** 2641–2650 (2013).
54. Essmann, U. *et al.* A smooth particle mesh Ewald method. *J. Chem. Phys.* **103,** 8577 (1995).
55. Bussi, G., Donadio, D. & Parrinello, M. Canonical sampling through velocity rescaling. *J. Chem. Phys.* **126,** 014101 (2007).
56. Parrinello, M. Polymorphic transitions in single crystals: A new molecular dynamics method. *J. Appl. Phys.* **52,** 7182 (1981).
57. Ferreira de Freitas, R. & Schapira, M. A systematic analysis of atomic protein-ligand interactions in the PDB. *Medchemcomm* **8,** 1970–1981 (2017).
58. Porollo, A. & Meller, J. Prediction-based fingerprints of protein-protein interactions. *Proteins* **66,** 630–645 (2007).
59. Eisenhauer, E. A. *et al.* New response evaluation criteria in solid tumours: revised RECIST guideline (version 1.1). *Eur. J. Cancer* **45,** 228–247 (2009).
60. Gorelick, A. Companion code for article "AKT mutant allele-specific activation dictates pharmacologic sensitivities. *Zenodo* (2021). doi:10.5281/zenodo.5111040

STUDY PROTOCOL
A Pilot study of AZD5363 for Patients with Advanced Solid Tumors Harboring Mutations in AKT1, AKT2, or AKT3

PROTOCOL FACE PAGE FOR
MSK THERAPEUTIC/DIAGNOSTIC PROTOCOL

Principal Investigator/Department:	Alison Schram, MD	Medicine
Co-Principal Investigator(s)/Department:	Alexander Drilon, MD	Medicine
Investigator(s)/Department:	Mrinal Gounder, MD<br>James J. Harding, MD<br>Bob Li, MD<br>Komal Jhaveri, MD<br>Gopakumar Iyer, MD<br><br>Elizabeth Won, MD<br>Parisa Momtaz, MD<br>Chau Dang, MD<br>Chung-Han Lee, MD, PhD<br>Min Yuen Teo, MD<br><br>Loren Michel, MD<br>Serena Wong, MD<br>Marina Shcherba, DO<br>Jason Konner, MD	Medicine
	Gloria Wasilewski, RN<br>Sherie Mar-Chaim, RN<br>Maureen Kennedy, RN<br><br>Raylene Langish, RN<br>Nicole Heinz, RN<br>Jacintha Ballingall, NP<br>Jessica Marchisotto, RN<br><br>Mary Kate Kasler, NP<br>Madeline Merrill, NP<br>Katherine Kargus, RN<br>Lauren Kaplanis, RN<br>Megan Troxel, RN<br>Alyson Merced, RN<br>Brittany Punzone, RN<br>Kellye Koubek, RN<br>Ryan Mitchell, RN<br>Kristen Clemens, RN<br>Kristen Stasi, RN	Nursing

	Dazhi Liu, Pharm D<br><br>Ines Nikolovski, MD<br>Heiko Schöder, MD<br><br>Joseph P. Erinjeri, MD, PhD<br><br>Alexia Iasonos, PhD	Pharmacy<br><br>Radiology<br><br>Interventional Radiology<br><br>Epidemiology and Biostatistics
Consenting Professional(s)/Department:	Mrinal Gounder, MD<br><br>Alexander Drilon, MD<br>James J. Harding, MD<br>Komal Jhaveri, MD<br>Gopakumar Iyer, MD<br>Alison Schram, MD<br>Bob Li, MD<br><br>Elizabeth Won, MD<br>Parisa Momtaz, MD<br>Chau Dang, MD<br>Chung-Han Lee, MD, PhD<br>Min Yuen Teo, MD<br><br>Loren Michel, MD<br>Serena Wong, MD<br>Marina Shcherba, DO<br>Jason Konner, MD<br><br>Mary Kate Kasler, NP<br>Jacintha Ballingall, NP<br>Madeline Merrill, NP	Medicine<br><br>Nursing

Please Note: A Consenting Professional must have completed the mandatory Human Subjects Education and Certification Program.

OneMSK Sites
Manhattan
Westchester
Monmouth

Memorial Sloan Kettering Cancer Center
1275 York Avenue
New York, New York 10065

Table of Contents

1.0 PROTOCOL SUMMARY AND/OR SCHEMA 6
2.0 OBJECTIVES AND SCIENTIFIC AIMS 8
2.1 Primary Objectives 8
2.2 Secondary Objectives 8
2.3 Exploratory Objectives 8
3.0 BACKGROUND AND RATIONALE 8
3.1 PI3K/Akt/mTOR Pathway 8
__3.1.1 Rationale for inclusion of non-E17K *AKT1, AKT2, and AKT3* mutations 9
3.2 AZD5363 Pre-clinical studies 11
3.2.1 Non-clinical information and correlative studies 12
3.3 Clinical information 14
3.4 Combination therapy with Fulvestrant (ER+ breast cancer patients only) 18
3.5 Combination therapy with enzalutamide in prostate cancer 20
3.6 Rationale for correlative studies 20
4.0 OVERVIEW OF STUDY DESIGN/INTERVENTION 20
4.1 Design 20
4.2 Intervention 20
5.0 THERAPEUTIC/DIAGNOSTIC AGENTS 21
5.1 Identity of investigational product: AZD5363 21
5.2 Drug supply and distribution 21
5.3 Dosing instructions 21
__5.3.1 All tumor types other than ER+ breast and prostate cancer patients 21
__5.3.2 ER+ breast cancer patients 21
__5.3.3 Prostate cancer patients 22
6.0 CRITERIA FOR SUBJECT ELIGIBILITY 22
6.1 Subject Inclusion Criteria 22
6.2 Subject Exclusion Criteria 23
7.0 RECRUITMENT PLAN 24
8.0 PRETREATMENT EVALUATION 25
9.0 TREATMENT/INTERVENTION PLAN 26
9.1 AZD5363 administration for all tumor types other than ER+ breast and prostate cancer patients 26

9.2 AZD5363 administration with fulvestrant therapy for ER+ breast cancer patients 26
9.3 AZD5363 administration with enzalutamide therapy for prostate cancer patients 27
9.4 Restrictions 27
9.5 Concomitant treatments 28
9.6 General Considerations 29
9.7 Use of Metformin 29
9.8 Study procedures for all patients 29
10.0 EVALUATION DURING TREATMENT/INTERVENTION 30
10.1 Study plan 30
10.2 Collection of plasma for analysis of cfDNA 31
10.3 Off Study Visit 31
11.0 TOXICITIES/SIDE EFFECTS 32
11.1 Toxicity Management: general considerations 32
11.2 Toxicity dose modifications 33
11.2.1 Toxicity dose modifications for AZD5363 33
11.2.2 Toxicity dose modifications for fulvestrant 333
11.2.3 Toxicity dose modifications for enzalutamide 33
11.3 Gastro-intestinal toxicity related to AZD5363 34
11.4 Glucose abnormalities related to AZD5363 34
11.5 Hepatotoxicity 36
11.6 Skin toxicity related to AZD5363 36
12.0 CRITERIA FOR THERAPEUTIC RESPONSE/OUTCOME ASSESSMENT 37
13.0 CRITERIA FOR REMOVAL FROM STUDY 38
14.0 BIOSTATISTICS 38
14.1 General Considerations 38
14.2 Primary Endpoint 38
14.3 Secondary Endpoints 38
15.0 RESEARCH PARTICIPANT REGISTRATION AND RANDOMIZATION PROCEDURES 41
15.1 Research Participant Registration 41
15.2 Randomization 41
16.0 DATA MANAGEMENT ISSUES 41
16.1 Quality Assurance 42
16.2 Data and Safety Monitoring 42

17.0 PROTECTION OF HUMAN SUBJECTS 42
17.1 Privacy 43
17.2 Serious Adverse Event (SAE) Reporting 43
17.2.1 SAE Reporting to AstraZeneca 44
17.2.2. Adverse Event Definition........ 45
18.0 INFORMED CONSENT PROCEDURES 46
19.0 REFERENCES 47
20.0 APPENDICES 49
Appendix A Concomitant Treatment Cautions and Restrictions for AZD5363
Appendix B RECIST v1.1
Appendix C Therapeutic Response assessment for Prostate Cancer patients (PCWG3)
Appendix D RANO
Appendix E Categorization of Quantitative Protein Urine Analysis
Appendix F Patient instructions for the management of Diarrhea

1.0 PROTOCOL SUMMARY AND/OR SCHEMA

This will be an open label, single institution, non-randomized, pilot study for patients with advanced solid tumors harboring mutations in AKT1, AKT2, or AKT3, to evaluate the anti-tumor efficacy of AZD5363.

For patients other than ER+ breast cancer and prostate cancer, AZD5363 will be administered orally at the single agent recommended Phase II dose on the intermittent schedule of 480mg twice daily for 4 days on, 3 days off dosing. One treatment cycle will comprise 28 days. ER+ breast cancer patients will receive AZD5363 at the combination recommended Phase II dose of 400 mg twice daily for 4 days on, 3 days off with fulvestrant 500mg IM on days 1, 15, 29 (or Cycle 2 Day 1) and then every 4 weeks. Prostate cancer patients will receive AZD5363 at a dose of 400 mg twice daily for 4 days on, 3 days off with enzalutamide 160 mg PO once daily continuously. The three cohorts (breast, prostate, other solid tumors) will be evaluated separately.

Patients will be evaluated at the start of every cycle for the first 24 weeks and every 2 cycles (approximately every 8 weeks) thereafter. Research bloods will be collected for cfDNA analysis at the time of every study visit. Tumor imaging studies will be obtained every 2 cycles (approximately 8 weeks) for the first 24 weeks and every 3 cycles (approximately 12 weeks) thereafter. Patients will remain on study treatment until progression of disease, intolerable adverse events, or withdrawal for any other reason.

Patients will also undergo optional serial tumor biopsies (pre-treatment and post-progression) for the purposes of identifying genomic mechanisms of de novo and acquired resistance as well as increased sensitivity to AZD5363.

Study Schema

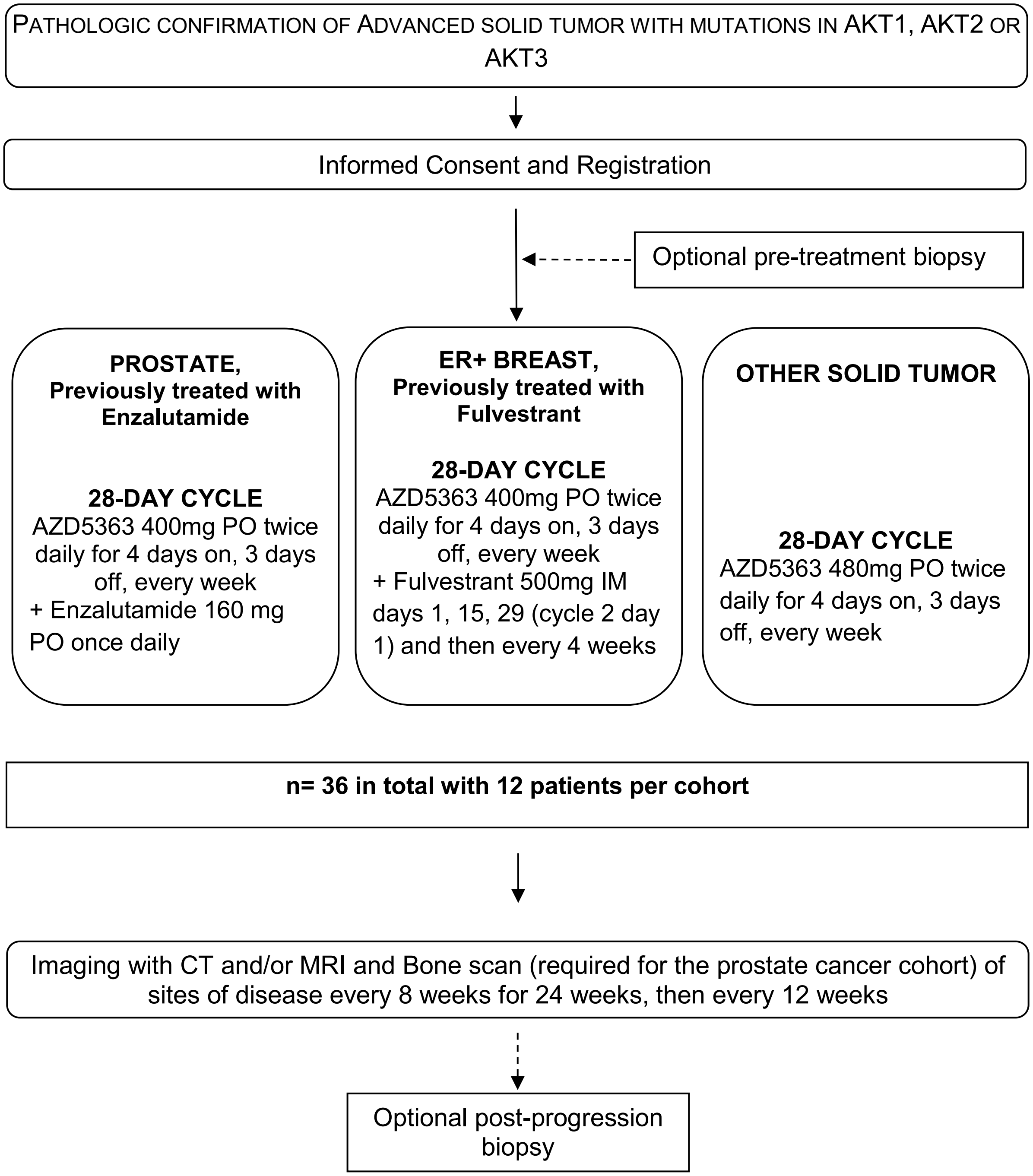

2.0 OBJECTIVES AND SCIENTIFIC AIMS

2.1 Primary Objectives

To determine the objective response rate (ORR) of AZD5363 in patients with advanced solid tumors harboring mutations in *AKT1*, *AKT2*, or *AKT3*, by evaluation of tumor response according to RECIST v1.1, PCWG3, RANO or prostate-specific antigen (PSA) measurement as applicable.

A response is defined as any of the following: a response according to RECIST v 1.1, PCWG3 (for patients with measurable visceral and/or nodal disease at baseline) or RANO as applicable **or** a reduction in the PSA level of 50% or more (for prostate cancer patients without visceral and/or nodal disease at baseline), with a confirmatory assessment at least 4 weeks later.

2.2 Secondary Objectives

- To assess the clinical benefit rate (CBR) (complete response, partial response, or stable disease) at 24 weeks according to according to RECIST v1.1, PCWG3, or RANO as applicable, in patients with advanced solid tumors harboring mutations in AKT1, AKT2, or AKT3.
- Progression-free survival (PFS) according to RECIST v1.1, PCWG3, or RANO as applicable
- Confirm tolerability of AZD5363
- Additional objectives for Prostate cohort:
  - Estimate the proportion of patients without PSA progression by PCWG3 criteria at 24 weeks
  - Determine the 12-week PSA RR
  - Evaluate PSA-PFS by PCWG3 criteria

2.3 Exploratory Objectives

- Examine the clonality and genetic configuration of the *AKT* sensitizing mutation in the pre-treatment specimens of study patients and their associated clinical response to AZD5363.
- Examine the pattern of co-mutated genes in *AKT*-mutant tumors and their association with treatment response or resistance
- Describe possible mechanisms of acquired resistance to AKT inhibition.

3.0 BACKGROUND AND RATIONALE

3.1 PI3K/Akt/mTOR Pathway

PI3K–AKT pathway dysregulation is a hallmark of various cancers, seen in up to 50% of all solid tumors.[1-3] Among other mechanisms, aberrant pathway activation can arise from somatic mutations in *PIK3CA*, *AKT1* or from loss/inactivation of the *PTEN* suppressor gene.[4-6] Consequently, significant therapeutic efforts have focused on developing inhibitors of various nodes in the PI3K signaling cascade with varying and often limited therapeutic success.[7-10]

Although *AKT1* mutations are among the least frequent of somatic events within the pathway, they are observed in a broad range of human cancers including those of the breast, colon, bladder, and ovary.[11-18] E17K is the most common mutational hotspot activating *AKT1* and promotes its pathological localization to the plasma membrane, thereby stimulating constitutive downstream signaling.[15,19] Indeed, *AKT1* E17K has recently been credentialed as an oncogenic driver and rational drug target in the ongoing phase 1 study of AZD5363 in patients with *AKT1*-mutant advanced solid tumors (NCT01226316), where most patients enrolled derived clinical benefit from targeted AKT inhibition, with some degree of tumor regression seen in >70% of patients (**Figure 1**).[20] These findings affirmed that targeting this central node (*AKT1*) is an attractive therapeutic modality in advanced cancers.

3.1.1 Rationale for inclusion of non-E17K *AKT1, AKT2, and AKT3* mutations

Ongoing enterprise-scale prospective tumor genotyping efforts at MSKCC provide a unique opportunity to expand the list of potential alterations in the AKT isoforms that could be targeted in a similar manner to *AKT1* E17K.[21,22] Analysis of both large-scale retrospective and our prospective, institutional, clinical sequencing data, across cancer types has shown that beyond the E17K hotspot, *AKT1* has a long tail of mostly private or low-incidence mutations that may be sensitizing to AKT inhibition.[23] Work pioneered in the Taylor lab within the Center for Molecular Oncology has established a computational framework that takes three complementary approaches to identify such long tail driver mutations, including recurrence, paralogy, and protein structure.[23] Applying these analytical tools has predicted both known and novel mutant residues in all three AKT isoforms that may be activating (**Figure 2a**). These results include private mutations in one isoform that become hotspots when paralogy is taken into account (such as W22). Beyond paralogous sequence analysis, two clusters of physically adjacent mutations in three dimensions have also been identified, one cluster defined by mutations [W22, E322, D323 (D324 in AKT2)] that lie at the PH-Kinase domain interface and directly abut *AKT1-3* E17K physically (within 5 angstroms; **Figure 2b**), and another larger group of rare mutations clustering with *AKT1* Q79K (Q78 in *AKT3*) including mutant residues P51, L52 (L51 in *AKT3*), F55, and W80 (**Figure 2c**). Furthermore, we have identified entirely novel recurrent in-frame insertions in the PH domain in both *AKT1* and *AKT2* in active patients in our prospective clinical cohort (**Figure 2d**). These insertions are flanked by two known activating *AKT1* hotspot mutations (L52 and Q79), reside in the PH-Kinase regulatory interface, and are likely novel activating mutations, a precedent for which exists in other actionable oncogenes such as *EGFR*, *ERBB2*, and *MAP2K1*. The culmination of this work has nominated candidate sensitizing mutations as follows: 1) novel low-incidence hotspots; 2) clonal non-hotspot mutations with evidence of mutations in paralogous residues across AKT isoforms; 3) mutations that cluster in protein structure in close proximity to known activating hotspots; 4) novel in-frame insertions in critical regulatory regions. Using these *highly selective and principled criteria*, we have selected 15 *AKT1-3* mutations and 28 mutant alleles already observed in the prospective clinical series at MSKCC. In total, these candidate driver mutations represent approximately 14% of the nearly 230 non-truncating, non-E17K variants detected in our prospective clinical sequencing series. **In fact, we are currently following 32 active patients with these highly selected low-frequency alterations**. In addition, we have identified 14 rare *AKT1-3* mutant residues/alleles observed in the large-scale retrospective dataset (primarily TCGA cohorts re-analyzed through our informatic pipeline), but not yet observed in MSKCC patients, that we will continue to monitor for trial enrollment (**Table 1**). Finally, in our clinical series we are following 38 non-breast/non-gynecologic *AKT1* E17K mutant patients.

We propose a pilot study of AZD5363 with the objective of treating patients with low-frequency variants identified by our analytic pipeline as potentially sensitizing to AKT inhibitor therapy. We would also propose treating non-breast/non-gynecologic cancer patients with *AKT1* E17K mutations given that the very heterogeneous Study 1(*#14-214: Phase I, Open-Label, Multicentre Study to Assess the Safety, Tolerability, Pharmacokinetics and Preliminary Anti-tumor Activity of Ascending Doses of AZD5363 under Adaptable Dosing Schedules in Patients with Advanced Solid Malignancies*) “D - Other” cohort, did not build a sufficiently large treatment experience in tumor types such as lung cancer, prostate cancer, and colon cancer, where the rate of AKT1 mutations is higher than previously appreciated. *This study will build on the success of the initial AKT1 E17K-focused program by potentially significantly expanding the number of genomic variants that could be targeted by AZD5363*. Preliminary analysis of the first 10,000 clinically sequenced cases at MSKCC suggest that non-*AKT1* E17K activating mutations, in aggregate, represent up to 25% of all activating *AKT* mutations observed. This represents a substantial portion of patients that may potentially benefit from AZD5363 and about which little is currently known. Moreover, we predict that as our genomic datasets expand in size, our analytic pipeline will identify additional AKT1/2/3 candidates for targeting in this manner.

Figure 1: Best percentage change from baseline waterfall Plot for AZD5363 Study 1 Part D (all tumor types)

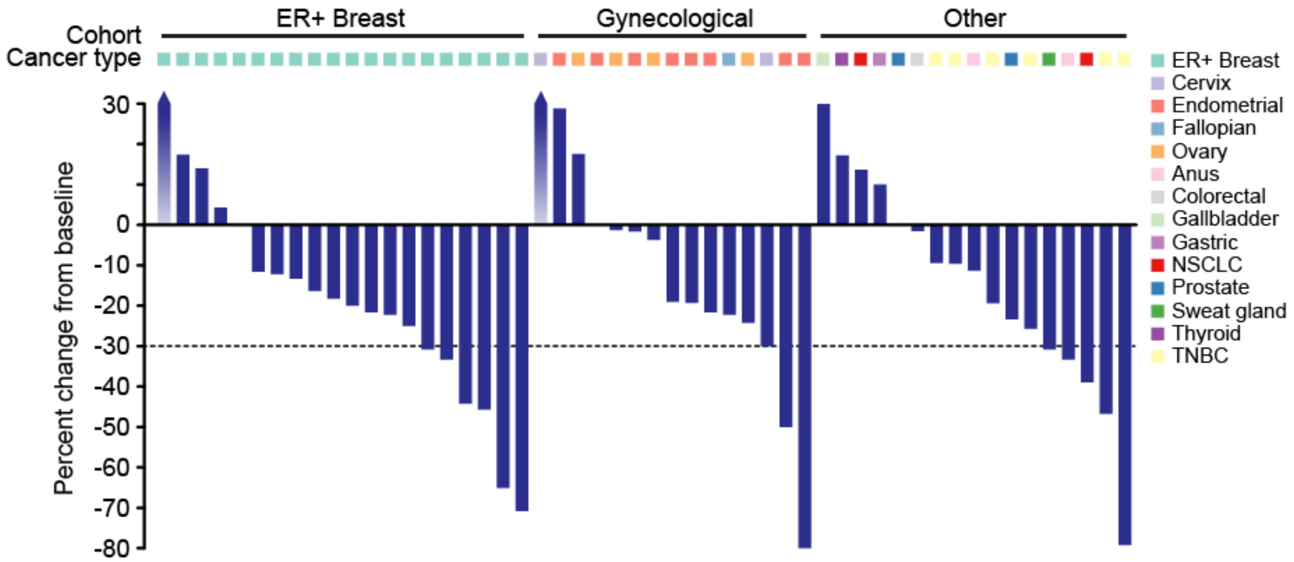

Data source: Analysis for manuscript submission October 2016

Table 1: Candidate Activating Mutations by Residue and Observed Tumor Type in MSKCC patients

Isoform	Codon	Mutants	Tumor types
AKT1	R15	Q	Cervix
AKT1,2,3	E17	K	Multiple
AKT1,2,3	W22	R,C	Lung
AKT1	E40	K	Kidney, colorectal, ovarian
AKT3	L51	R	Lung carcinoid
AKT1	L52	R/H	Breast, kidney
	F55	Y	Breast

AKT3	Q78	K	Prostate
AKT1	Q79	K	Endometrial, prostate, AML
	W80	R	Endometrial, prostate, colorectal
AKT3	R247	H,C	Lung, glioma, skin
	R249	H,S	Bladder, lung, colorectal
AKT1	D323	G,Y	Bladder, lung, prostate, kidney
AKT2	D324	G,H,N	Colorectal, AML, kidney
AKT1	R370	C,H	Colorectal, glioma

Figure 2: Visualizing Candidate Drivers in AKT1, AKT2, and AKT3

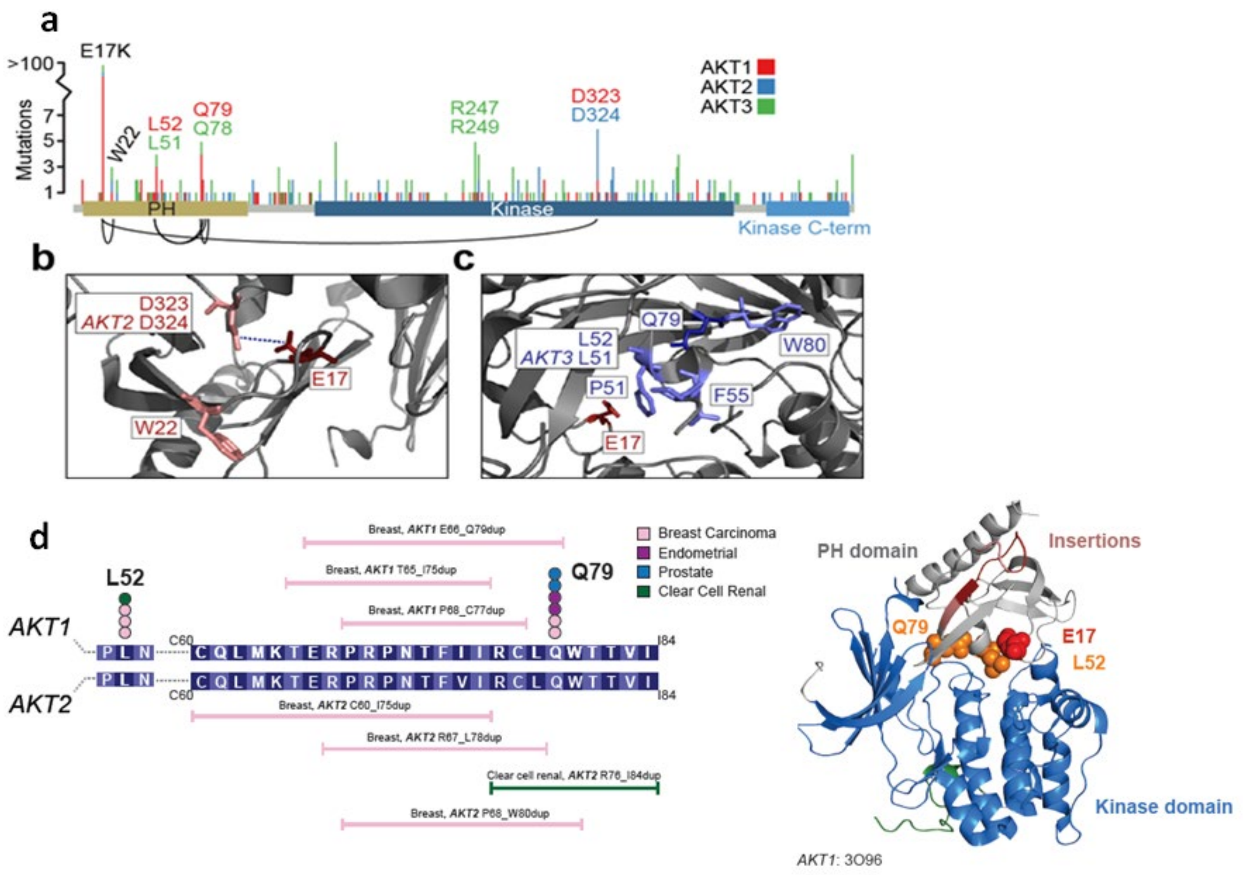

3.2 AZD5363 Pre-clinical studies

AZD5363 is a potent, selective inhibitor of the kinase activity of the serine/threonine AKT/PKB (protein kinase B) that is fully described in its Investigator Brochure.

In brief, AKT is part of the AGC family of kinases. Mammalian cells express three closely related AKT isoforms: AKT1 (PKBα), AKT2 (PKBβ) and AKT3 (PKBγ), all encoded by different genes. AKT is a node of multiple signaling pathways promoting tumorigenesis, inhibiting apoptosis, impacting on cell cycle and promoting invasion and migration.

The PI3K/AKT/PTEN pathway is frequently deregulated in cancer and drives tumor growth and cell survival.[24] All 3 AKT isoforms are activated in different tumor types including breast,

prostate, ovarian, pancreatic and gastric cancers, and this activation is often associated with resistance to established cancer therapies as well as advanced disease and/or poor prognosis.[25] AKT activation in tumors is largely due to input from other signaling pathways upstream of AKT (e.g. mutation of oncogenes such as Ras, Bcr-abl, mutation of receptor tyrosine kinases such as EGFR, amplification of Her2, loss of PTEN function, mutations of PI3K).

Inhibitors of AKT are anticipated to have efficacy when dosed in combination with cytotoxic chemotherapies or in combination with targeted or antihormonal agents. AZD5363 inhibits all three AKT isoforms (AKT1, AKT2 and AKT3) and therefore has the potential to provide clinical benefit over a range of therapeutic indications and is in development for the treatment of patients with cancer.

3.2.1 Non-clinical information and correlative studies

AZD5363 is a potent inhibitor of AKT 1, 2 and 3 (half maximal inhibitory concentration of the drug [IC_{50}] <10 nM). Non-clinical *in vitro* and *in vivo* assays have demonstrated inhibition of phosphorylation of the AKT substrates GSK3β and PRAS40, tumor cell proliferation and xenograft tumor growth models. With treatment of nude mice bearing BT474c xenografts there was time- and dose-dependent inhibition of phosphorylation of the AKT substrates PRAS40 and GSK3β, and the downstream biomarker S6, after single oral doses of 100 and 300 mg/kg. Chronic oral treatment of nude mice bearing a variety of established and primary xenografts with AZD5363 resulted in dose-dependent tumor growth inhibition.

Further details are provided in the Investigators' Brochure.

3.2.2 PK drug-drug interactions

In vivo experiments indicate that AZD5363 is a time-dependent inhibitor of CYP3A4, which may result in increased exposure of drugs metabolized via CYP3A4 and with the potential to increase the toxicity of these drugs when coadministered with AZD5363. AZD5363 is itself a substrate of CYP3A4 although data available to date suggests that glucuronidation may be the major metabolic route. Coadministration of CYP3A4 inhibitors may increase exposure to AZD5363 and hence potentially affect efficacy/toxicity, and hence increase the risk of time-dependent inhibition (and resultant toxicity of CYP3A4 substrates). In addition, coadministration of CYP3A4 inducers may decrease the exposure to AZD5363 and may potentially affect efficacy. AZD5363 is also a moderate inhibitor of CYP2D6 *in vitro*. This may increase the exposure of drugs metabolized via CYP2D6 with the potential to increase the toxicity of these drugs when co-administered.

Use of potent inhibitors or inducers of CYP3A4 within 2 weeks before the first dose of study treatment (3 weeks for St John's Wort) should be avoided. All patients should avoid concomitant use of drugs, herbal supplements and/or ingestion of foods known to potently modulate CYP3A4 enzyme activity from 2 weeks before first dose until 2 weeks after the last dose of study treatment. All patients should avoid concomitant use of drugs and herbal supplements known to be CYP3A4 or CYP2D6 substrates from 2 weeks before first dose until 2 weeks after the last dose of study treatment wherever possible. If co-administration is necessary for appropriate clinical care then additional monitoring for signs of toxicity related to increased exposure to the substrates may be required. A list of concomitant treatment cautions and restrictions are provided in Appendix A.

Emerging *in vitro* data has revealed that AZD5363 has a potential to inhibit the OATP1B1 transporter. This transporter is implicated in the distribution and clearance of many of the statins. Of the statins that are minimally affected by CYP3A4 inhibition, rosuvastatin and pravastatin (but not fluvastatin) can be affected by OATP1B1 inhibition. In an assessment of the potential for AZD5363 to inhibit OATP1B1 based on the *in vitro* signal, the AUC of these drugs may be increased by 1.3-fold for pravastatin and 1.5-fold for rosuvastatin (static assessment based on maximal free liver inlet concentration of AZD5363). As a conservative response to this emerging data it is recommended that doses of rosuvastatin be capped to 10 mg once daily and pravastatin to 40 mg once daily when combined with AZD5363, and for a 2-week period before and after AZD5363 treatment.

Further details are provided in the Investigators' Brochure.

3.2.3 Preclinical Safety

Potential risks identified non-clinically with AZD5363
This section is based upon non-clinical toxicology and safety pharmacology studies with AZD5363. The toxicological profile of AZD5363 has been evaluated in rats and dogs in studies of up to 9 months in duration. The key risks identified from non-clinical assessments to date are considered to be effects on glucose homeostasis and insulin signaling, cardiac function, and renal function. Other tissues affected in non-clinical studies have included the reproductive organs, liver, hypothalamic-pituitary axis and hematopoietic system. In addition, geno toxicity and phototoxicity have been assessed.

Further information is in the Investigator Brochure.

Glucose homeostasis and insulin signaling: Marked increases in blood insulin and glucose levels were noted following dosing in the dog and rat studies. Levels of insulin and glucose had generally returned to normal 24 hours post-dose. AKT is known to be involved in the regulation of glucose metabolism and therefore these changes are likely to be related to the primary pharmacology of AZD5363.[26]

Cardiac function: AZD5363 inhibits the hERG channel, with an IC_{50} of 73.0 mol/L, via an underlying mechanism that may be due to increases in plasma glucose and insulin levels.[27,28] Data from the isolated rabbit heart model suggest that AZD5363 may be classified as low risk in terms of triggering the QT-associated arrhythmia of Torsades de Pointes. Increases in cardiac contractility have been observed in some non-clinical models; the mechanism underlying these observations is unknown. Decreases in blood pressure have also been noted, consistent with the vaso-relaxant activity of AZD5363 identified in a rat aorta study and the increase in coronary flow seen in the isolated rat and rabbit heart studies. It is possible that the vasoactive effects of AZD5363 occur via inhibition of ROCK1, which is known to play an important role in regulation of vascular tone.[29]

Renal function: Polyuria, glucosuria, proteinuria, increased water consumption, and decreased tubular epithelial cell size have been observed non-clinically, but there was no associated histopathology in the kidney. These effects are consistent with the pharmacological activity of AKT in terms of a role in proximal tubular glucose and phosphate transport.[30]

Reproductive toxicity: Studies in rats and dogs showed degenerative changes in the testes and epididymis in males, and decreased prostate weights. Findings in the female reproductive organs (uterus and ovaries) were noted in the 1-month rat toxicity study, but were limited to a

reversible reduction in organ weight with no histopathological findings. AZD5363 had an adverse effect on embryonic survival, plus early postnatal growth when administered to pregnant and lactating rats. Exposure to AZD5363 was confirmed in suckling pups, which may indicate the potential for excretion of AZD5363 in milk.

Further information is in the Investigator Brochure.

3.3 Clinical information

As of the most recent data cut-off date (04 Oct 2016) for the Investigator Brochure (14 Dec 2016), 5 AstraZeneca (AZ) sponsored Phase 1 and Phase 2 studies have been conducted or are ongoing, with data from approximately 360 patients collected. In addition, 18 investigator sponsored studies are planned or have recently commenced recruitment.

The single agent MTD for oral AZD5363 administered twice daily has been defined at 480mg given on the intermittent schedule of 4 days on, 3 days off dosing.

AZD5363 is also being evaluated in several ongoing early phase combination studies with paclitaxel, docetaxel, olaparib, fulvestrant and ezalutamide.

3.3.1 Phase I experience: Advanced solid tumors

In general, the AZ-sponsored studies have been primarily designed to determine the MTDs and recommended doses for Phase 2 for the various proposed AZD5363 dosing schedules. As such, the studies have recruited patients from advanced cancer populations that have been heavily pre-treated and who show resistance to a number of prior therapies. None of the dose-finding parts of the studies presented selected for patients whose tumors harbor *PIK3CA* or *AKT* mutations. However, tumor response data are available from 131 patients from Studies D3610C00001 (Parts A and B) and D3610C00004. These results show that 3 of the 131 patients had RECIST partial responses (PRs): 1 whose tumor was positive for *PIK3CA* mutation and 2 where the tumor tested positive for *AKT1* mutation. Tumor mutation status was not systematically determined in these Phase 1 studies.

The expansion parts of Study D3610C00001 (Parts C and D) recruited an advanced cancer population selected for patients with proven *PIK3CA* (Part C) or *AKT1* (Part D) mutations.

In patients with *PIK3CA* -mutation positive tumors (Part C), 1 ER+ breast cancer patient and 2 gynecological cancer patients had a confirmed RECIST partial response (PR).

In patients with *AKT1* E17K-mutation positive tumors (Part D), partial responses were observed in 20% of ER+ breast (4/20) and gynecologic (3/15) cancers, with additional responses observed in triple negative breast and non-small cell lung cancer patients (one each). In *AKT1* non-E17K patients, tumor regressions not meeting response criteria were observed in two *AKT1* Q79K-mutant patients (prostate and ovarian), including one lasting 14 months. The sponsor of the D3610C00001 study, AstraZeneca, decided that the efficacy of AZD5363 monotherapy, primarily in ER+ breast cancer which represented nearly 50% of enrollment to this cohort (Part D) and was their primary development focus, was insufficient to seek breakthrough therapy designation (although the confirmed ORR was 20% and the

median PFS 5.5 months for ER+ breast cancers enrolled to this cohort). As a result, Part D (D3610C00001/#14-214), which enrolled for the most part AKT1 E17K mutant tumors only to receive AZD5363 monotherapy has been closed and AstraZeneca have amended the D3610C00001 study to include a fulvestrant combination (Part E) for ER+ breast cancers. Despite this, the confirmed ORR in the “Other solid tumor” cohort of Part D was 18% (3/17) in very heavily pretreated patients (median prior systemic therapies = 4, range 1-12) including a PR observed in an *AKT1* E17K mutant lung cancer patient lasting over a year. Moreover, 70% (12/17) of the “Other solid tumor” patients overall had some degree of tumor regression (not meeting RECIST criteria) in response to AZD5363. Furthermore, many tumor types were enrolled only once to this “Other solid tumor” cohort and therefore it represents an insufficient dataset to make any definitive determination of AZD5363 efficacy in other solid tumors in this genomically selected context and as such warrants further study in this proposed pilot study.

Further expansion cohorts (Parts E and F of D3610C00001/#14-214) of AZD5363 combined with Fulvestrant are ongoing in ER+ breast cancer patients with *AKT1* E17K mutations and PTEN alterations respectively. Within Parts E and F of #14-214, there are fulvestrant exposed/resistant (Er and Fr) and fulvestrant naïve/delayed (Ed and Fd) cohorts. Er has reached target accrual internationally and is currently closed to enrollment, Ed continues to enroll fulvestrant naïve ER+ breast cancer patients harboring an *AKT1* E17K mutation.

3.3.2 Clinical Safety

Possible risks identified clinically with other AKT inhibitors

There are currently several AKT inhibitors in clinical development and the safety profile of AKT inhibitors is emerging.[31] Common drug-related AEs emerging from published clinical trials included skin disorders (rash), gastrointestinal symptoms (diarrhea, nausea), fatigue, and musculoskeletal pain. [32]

Data from clinical studies with AZD5363

Overview

Over 360 patients have been treated with AZD5363 as either monotherapy or in combination therapy with paclitaxel. Of these 5 studies, 2 are ongoing, 2 have completed (Studies D3610C00004 and D3610C00007), and 1 (Study D3610C00003, a monotherapy study in late line unselected CRPC population) has been terminated early following an unscheduled interim analysis that indicated that AZD5363 monotherapy was unlikely to generate a positive efficacy signal in a heavily pre-treated and unselected advanced prostate cancer population.

The AEs of hyperglycemia, rash, diarrhea, hypersensitivity, stomatitis, dry skin, and pruritus are expected for AZD5363. Additional AEs that are commonly reported for AZD5363, (those affecting >30% in the pooled monotherapy intermittent group), irrespective of causality, are decreased appetite, nausea, vomiting, and fatigue. Patients enrolled in the AZD5363 clinical studies have significant comorbidities, multiple concomitant medications, and have been exposed to other anticancer treatment before receiving AZD5363.

Hyperglycemia: Hyperglycemia (defined in the clinical studies as at least 1 postbaseline laboratory report of a pooled glucose value [non-fasting and fasting values combined] >ULN) is a frequent clinical observation, but is transient and reversible on cessation of treatment. To

date, the overall incidence of hyperglycemia is 95.6% in the intermittent monotherapy 480 mg BD 4 days on; 3 days off dosing schedule, 100% in the intermittent 400 mg BD 4 days on; 3 days off combination with paclitaxel schedule, and 92.3% in the intermittent 400 mg BD 4 days on; 3 days off combination with fulvestrant schedule. The majority of cases occurred within the first week of study treatment (88.5% in patients treated with AZD5363 monotherapy 480 mg BD intermittent dosing, 100% in patients treated with AZD5363 400 mg BD intermittent with paclitaxel, and 84.6% in patients treated with AZD5363 400 mg BD intermittent with fulvestrant). Across these 3 dosing schedules, the proportion of patients who received metformin for elevated glucose levels ranged from 0% (intermittent dosing with paclitaxel), to 31.7% (AZD5363 intermittent monotherapy).

Consistent with the observed transient hyperglycemia associated with AZD5363 Cmax, it has now become apparent that there is a population trend to increasing HbA1c on AZD5363. Examination of all monotherapy, intermittent dosed patients at 400 mg and above reveals an increase in mean HbA1c of 9.27 mmol/mol (90% CI: 7.50, 11.03) at 12 weeks (i.e., an increase to 46.73 mmol/mol from the mean baseline value). A total of 156 patients from Study 1, Parts A to D, Study 3, Study 4 and Study 7, with both baseline and postbaseline measures were included in the analysis. The mean (SD) HbA1c at baseline for the population considered here was 37.5 (5.79) mmol/mol with a range from 24 to 60 mmol/mol. It appears that the majority of any HbA1C rise occurs during the first 12 weeks on treatment and that there is little subsequent elevation in the time period on drug beyond 12 weeks. The clinical relevance of this finding in the context of the populations and diseases under study is unknown. Additional analysis of HbA1c trends will be examined in the placebo-controlled data following unblinding of Study D3610C00002 (BEECH).

Rash: Rash is a frequent clinical observation. To date, the overall incidence of rash (an SMQ term including the preferred terms of rash, erythema, rash erythematous, rash macular, rash maculo-papular, rash papular, rash pruritic) is 46.4% in the intermittent monotherapy 480 mg BD 4 days on; 3 days off dosing schedule, 57.1% in the intermittent 400 mg BD 4 days on; 3 days off combination with paclitaxel schedule, and 26.9% in the intermittent 400 mg BD 4 days on; 3 days off combination with fulvestrant schedule. The proportion of patients with an AE of rash of CTCAE Grade 3 or above was 16.9%, 14.3%, and 11.5%, respectively. In the intermittent monotherapy 480 mg BD 4 days on; 3 days off dosing schedule, 39.3% of patients had a rash that was considered to be causally related to AZD5363. Seven patients (3.8%) had an SAE of rash, and 7 (3.8%) discontinued AZD5363 treatment due to rash. A total of 51 patients (27.9%) received treatment for rash.

Diarrhea: Diarrhea is a frequent clinical observation. To date, the overall incidence of diarrhea (relating to the SMQ term diarrhea) is 79.8% in the intermittent monotherapy 480 mg BD 4 days on; 3 days off dosing schedule, 85.7% in the intermittent 400 mg BD 4 days on; 3 days off combination with paclitaxel schedule, and 57.7% in the intermittent 400 mg BD 4 days on; 3 days off combination with fulvestrant schedule. The proportion of patients with an AE of diarrhea of CTCAE Grade 3 or above was 16.9%, 28.6%, and 3.8%, respectively.
In the intermittent monotherapy 480 mg BD 4 days on; 3 days off dosing schedule, 74.3% of patients had an AE of diarrhea that was considered to be causally related to AZD5363. Twelve patients (6.6%) had an SAE of diarrhea, and 2 (1.1%) discontinued AZD5363 treatment due to diarrhea. A total of 108 patients (59.0%) received treatment for diarrhea.

Hypersensitivity: Hypersensitivity is a commonly reported clinical observation being reported in 4 patients on the intermittent monotherapy dose of 480 mg BD 4 days on; 3 days off, and for 1 patient on the intermittent 400 mg BD 4 days on; 3 days off combination with paclitaxel

schedule. Symptoms of hypersensitivity included rash in association with 1 or more of AEs of the following AEs: flushing, pruritus, urticaria, throat itchiness, pyrexia, and facial and/or lip edema. In all patients, hypersensitivity or related AEs were considered causally related to AZD5363, and in 4 patients the reported AEs were considered serious, leading to hospitalization or prolonged hospitalization. One patient had a reported history of allergy to heat and cold. In all patients, the symptoms resolved with AZD5363 discontinuation and treatment with antihistamines and steroids. There were no AEs of hypersensitivity in the intermittent 400 mg BD 4 days on; 3 days off combination with fulvestrant schedule.

Stomatitis: Stomatitis is a very common clinical observation, affecting 32 patients (14.0%) in AZD5363 intermittent monotherapy, 10 patients (26.3%) in combination with paclitaxel, and 2 patients (7.7%) in combination with fulvestrant. In AZD5363 intermittent monotherapy, the majority of stomatitis AEs were Grade 1 or 2 (91.4%), and the maximum severity of AEs experienced by patients was CTCAE Grade 3 (8.6%). There were 3 AEs leading to dose interruption, and of these, all resulted in a positive de-challenge, and in the 2 patients subsequently re-administered the study drug, both received a reduced AZD5363 dose and both experienced negative re-challenge. The median duration of all AEs was 15 days.

Dry skin: Dry skin is a very common clinical observation, affecting 27 patients (11.8%) in AZD5363 intermittent monotherapy, 6 patients (15.8%) in combination with paclitaxel, and 1 patient (3.8%) in combination with fulvestrant. In AZD5363 intermittent monotherapy, the majority of dry skin AEs were Grade 1 (92.9%), and the maximum severity of AEs experienced by patients was CTCAE Grade 2 (7.1%). There were no AEs leading to dose interruption, reduction, or study discontinuation. The median duration of all AEs was 91.5 days.

Pruritus: Pruritus is a common clinical observation, affecting 21 patients (9.2%) in AZD5363 intermittent monotherapy group, 7 patients (18.4%) in combination with paclitaxel, and none in combination with fulvestrant. In AZD5363 intermittent monotherapy, the majority of pruritus AEs were CTCAE Grade 1 or 2, and 1 AE was Grade 3. There were no AEs leading to dose interruption or study discontinuation. The median duration of all AEs was 37 days.

AE profile for combination studies with AZD5363

AZD5363 has also been evaluated in investigator sponsored studies in combination with docetaxel, fulvestrant, enzalutamide and olaparib. The AE profile in all these studies was consistent with the individual AE profiles of each drug and with no additional observations in relation to safety and tolerability.

3.3.3 Pharmacokinetics

Multiple dose kinetics were consistent with single dose data and AZD5363 exposure was approximately dose-proportional over the investigated range of 80 to 800 mg. AZD5363 exposure was similar in Japanese and Western patients. The PK exposure following administration of AZD5363 via the tablet formulation was comparable to that following the same dose and schedule of the capsule formulation, supporting the switch to the tablet formulation for ongoing and future studies. The effects of food indicate that food may delay and reduce the rate of absorption of AZD5363, but the extent of absorption appeared to be comparable. The clinical relevance of this food effect is currently unknown, but a conservative approach has been taken to recommend that the existing food restrictions are maintained (i.e., patients to fast from 2 hours before dosing to 1 hour after dosing, where possible) in ongoing clinical studies.

Further details are provided in the Investigators' Brochure.

3.4 Combination therapy with Fulvestrant (ER+ breast cancer patients only)

Fulvestrant is an estrogen receptor antagonist approved and indicated for the treatment of hormone receptor positive metastatic breast cancer in postmenopausal women, supplied as an injection for intramuscular administration. "FAKTION", a phase 1b/2 randomized placebo controlled trial of fulvestrant +/- AZD5363 in postmenopausal women with advanced breast cancer previously treated with a third generation aromatase inhibitor is an ongoing clinical study (NCT01992952). FAKTION has established a combination MTD for fulvestrant and AZD5363 to be 400mg bid po in 4 days on – 3 days off schedule. The randomized Phase 2 component of FAKTION is ongoing using the 400mg AZD5363 dose. The FAKTION study tests the combination in an "all comer" (i.e. non-genomically selected) ER+ advanced breast cancer patient population.

It has recently been observed that suppression of PI3K pathway signalling results in induction of ER-dependent transcriptional activity, including increased expression of genes containing ER-binding sites and increased occupancy by the ER of promoter regions of upregulated genes. Additionally expression of ER mRNA and protein were also increased following PI3K pathway inhibition. The findings have been confirmed in animal model xenografts, patient-derived animal models, and in tumors from patients undergoing treatment with a PI3K alpha inhibitor, as well as an AKT inhibitor (AZD5363). These results suggest that PI3K pathway blockade in ER-positive breast cancer results in an ER-dependent transcriptional program that may be reversed with anti-hormonal therapies, and that simultaneous blockade of the PI3K pathway and ER signalling may be needed for optimal treatment of ER-positive breast tumors with over-activation of the PI3K pathway.[33,34]

On the basis of the biological evidence above and given the established combination dose for AZD5363 and fulvestrant, it is considered biologically and clinically appropriate to explore AZD5363 in combination with fulvestrant therapy where fulvestrant is considered to be a background therapeutic modality in ER positve advanced or metastatic breast cancer patients whose tumors harbour *AKT1,AKT2 or AKT3* mutations and who have exhibited prior fulvestrant resistance.

3.5 Combination therapy with enzalutamide in prostate cancer

Enzalutamide is an androgen receptor inhibitor approved and indicated for the treatment of metastatic Castration-Resistant Prostate Cancer (mCRPC).[35] RE-AKT, 'A randomised Phase II study of Enzalutamide (MDV3100) in combination with AZD5363 in Patients with Metastatic Castration - Resistant Prostate Cancer' (EudraCT Number: 2013-004091-34) is co-sponsored by The Royal Marsden NHS Foundation Trust and The Institute of Cancer Research. The study comprises of a safety run-in to establish a recommended phase 2 dose, a 1:1 randomised Phase 2 evaluating the antitumor activity of AZD5363 + enzalutamide vs placebo + enzalutamide, and a single stage Phase 2 expansion cohort exploring the utility of the combination to reverse resistance to enzalutamide. The safety run-in has established the RP2D to be 400mg bd AZD5363 (4days on / 3 days off) + 160mg enzalutamide OD. The AE profile observed in the Phase I part of RE-AKT was consistent with the individual AE profiles of each drug. The randomised Phase 2 part of the study commenced in July 2016 using the

400mg BD dose of AZD5363. The RE-AKT study tests the combination in an “all comer” (i.e. non-genomically selected) advanced prostate cancer patient population.

Inhibition of the PI3K/AKT/ mammalian target of rapamycin (mTOR) pathway has been shown to participate in a reciprocal feedback loop with the androgen receptor.[36] Inhibiting the PI3K/AKT/mTOR pathway results in activation of androgen receptor, whereas inhibition of androgen receptor signaling causes an activation of AKT. Therefore, we anticipated that combination of AZD5363 with an androgen receptor inhibitor such as enzalutamide (MDV-3100) would result in a greater therapeutic effect than the monotherapy of either agent. Enzalutamide enhanced the growth inhibitory effect of AZD5363 in both cell lines, and the combination of AZD5363 and enzalutamide was synergistic compared with AZD5363 alone in both cell lines.

In non-clinical studies, oral treatment of castrated nude mice bearing LNCaP prostate cancer xenografts with AZD5363 in combination with the androgen receptor antagonist enzalutamide (Xtandi®) showed enhanced anti-tumor activity compared with monotherapy treatments only. The combination also resulted in a sustained decline of serum PSA concentration.
Moreover, there is an ongoing randomized phase II study evaluating this hypothesis with another AKT inhibitor that is in clinical development along with an androgen synthesis inhibitor. Co-inhibition of AKT with Ipatasertib (GDC-0068), a potent novel oral ATP-competitive inhibitor of AKT, and of AR by abiraterone is being tested in patients with metastatic castration-resistant prostate cancer (mCRPC) after docetaxel chemotherapy (A. MARTIN Study). Interim analysis presented at ASCO 2016 showed that the combination may improve PFS and OS in mCRPC post docetaxel, with potentially increased benefit in patients with decreased PTEN expression. (De Bono J et al, J Clin Oncol 34, 2016 (suppl; abstr 5017)) Updated results presented by de Bono et al at ESMO 2016, confirmed an improved rPFS in patients with mCRPC with PTEN loss receiving combination AR and AKT inhibition therapy (11.5 months versus 4.6 months in those receiving AR inhibition alone, HR .39).

On the basis of the biological evidence above and given the established combination dose for AZD5363 and enzalutamide, it is considered biologically and clinically appropriate to explore AZD5363 in combination with enzalutamide therapy in metastatic Castration-Resistant Prostate Cancer (mCRPC) patients whose tumors harbour *AKT1,AKT2 or AKT3* mutations and who have exhibited prior enzalutamide resistance.

3.6 Rationale for correlative studies

The correlative studies in this trial are intended to establish the genomic determinants of AZD5363 sensitivity in AKT-mutant tumors through an examination of the clonality and genetic configuration of AKT sensitizing mutations, their co-mutational pattern and associated clinical response to AZD5363.

Analyses of post-progression biopsies are intended to determine mechanisms of resistance to AZD5363. ddPCR analyses of cfDNA will permit tracking of the specific AKT mutation found in each tumor and NGS analyses of cfDNA will permit us to observe the emergence of new somatic alterations in longitudinally acquired cfDNA and permit a comparison with post-progression biopsies in those patients that consent to the biopsies.

The genomic analyses performed on acquired specimens will include next generation sequencing (NGS) with the MSKCC IMPACT assay and whole exome sequencing (WES).

4.0 OVERVIEW OF STUDY DESIGN/INTERVENTION

4.1 Design

This will be an open label, single institution, non-randomized, pilot study. The primary objective is to determine the objective response rate to treatment with AZD5363 in patients with advanced solid tumors harboring mutations in *AKT1, AKT2, or AKT3*, by evaluation of tumor response according to RECIST v1.1, PCWG3, RANO or prostate-specific antigen (PSA) measurement as applicable.

4.24.2 Intervention

For patients with tumor types other than ER+ breast cancer and prostate cancer, AZD5363 will be administered orally at the single agent recommended Phase II dose on the intermittent schedule of 480 mg twice daily for 4 days on, 3 days off dosing. 1 cycle will be 4 weeks. Treatment will continue until progression, intolerable adverse events or withdrawal. To align the treatment of ER+ breast cancer patients enrolled to this study with the treatment of this tumor type in Study 1(#14-214: Phase I, Open-Label, Multicentre Study to Assess the Safety, Tolerability, Pharmacokinetics and Preliminary Anti-tumor Activity of Ascending Doses of AZD5363 under Adaptable Dosing Schedules in Patients with Advanced Solid Malignancies), ER+ breast cancer patients will receive AZD5363 at 400 mg twice daily for 4 days on, 3 days off + fulvestrant 500mg IM days 1, 15, 29 (or Cycle 2 Day 1) and then every 4 weeks. There will be no confounding effect due to the use of this combination, as all patients will have previously received fulvestrant. ER+ breast cancer patients must be post-menopausal as defined in section 6.1.

Prostate cancer patients treated on this study will similarly receive AZD5363 at 400 mg twice daily for 4 days on, 3 days off plus enzalutamide 160 mg PO once daily. Prostate cancer patients will have previously received enzalutamide.

The treatment plan is illustrated in the schema (Section 1.0) and described in Sections 9 and 10.

5.0 THERAPEUTIC/DIAGNOSTIC AGENTS

5.1 Identity of investigational product: AZD5363

The investigational product will be supplied by AstraZeneca. Additional information about the investigational product may be found in the Investigators' Brochure.

Labels will be prepared in accordance with Good Manufacturing Practice (GMP) and local regulatory guidelines. The labels will fulfill GMP Annex 13 requirements for labeling. Labeling will be performed by the MSKCC pharmacy at 53rd Street.

Study drugs will be kept in a secure place under appropriate storage conditions.

5.2 Drug supply and distribution

AstraZeneca will supply the investigational product (AZD5363) as a solid oral formulation (tablets).

Prior to April 2019:

Identity of investigational product

Investigational product	Form	Dosage strength	Manufacturer
AZD5363	Tablet	80mg, 160mg and 200mg	AstraZeneca

AZD5363 in a solid oral formulation will be supplied in white high density polyethylene (HDPE) child resistant bottles.

After April 2019:

As of April, 2019, AstraZeneca will be transitioning all studies of AZD5363 to a new formulation. The new drug product is presented for oral administration as a plain beige film-coated tablets containing 160 mg (round, 10 mm) or 200 mg (caplet shaped, 14 mm x 7 mm) of Capivasertib (AZD5363). Tablets are packed in high-density polyethylene (HDPE) bottles. Bottles are secured with a child-resistant closure; induction sealed membranes provide tamper evidence.

AstraZeneca will supply the tablets in both 60 tablet count and 76 tablet count bottles for both 200mg and 160mg tablets. The 76 tablet count bottle of 160mg is sufficient to supply patients receiving 480mg BID, 4 days on, 3 days off every week for an entire 21 day cycle.

The 1st dose reduction will still be 320mg, made up using 2 x 160mg tablets.

The 2nd dose reduction will need to be 200mg, previously it may have been 240mg, however there will be no tablet combinations available to make a 240mg dose, hence the 200mg will be the 2nd reduction going forward. This will be the 2nd dose reduction in the AstraZeneca sponsored phase III programme going forward.

5.3 Dosing instructions

5.3.1 All tumor types other than ER+ breast and prostate cancer patients

AZD5363 will be administered orally at the dose of 480mg, twice daily on an intermittent dosing schedule of 4 days on followed by 3 days off dosing.

5.3.2 ER+ breast cancer patients

Patients will receive AZD5363 400 mg twice-daily, 4 days on 3 days off dosing as tablets, commencing on day 1. Fulvestrant will be administered according to its approved dose of 500 mg intramuscularly on days 1, 15, 29 (or Cycle 2 Day 1) and once monthly thereafter.

5.3.3 Prostate cancer patients

Patients will receive AZD5363 400 mg twice-daily, 4 days on 3 days off dosing as tablets, commencing on day 1. Enzalutamide will be administered according to its approved dose of 160 mg PO once daily.

Twice daily doses should be taken at approximately the same time each morning and evening approximately 12 hours apart and taken with water in a fasted state from at least 2 hours prior to the dose to at least 1 hour post-dose.

Should a patient miss a scheduled dose, the patient will be allowed to take the dose up to a maximum of 2 hours after the scheduled dose time. If greater than 2 hours after the scheduled dose time the missed dose should not be taken and the patient should take their allotted dose at the next scheduled time. If a patient needs to take the dose earlier for whatever reason, the patient can take the dose up to 2 hours earlier than the scheduled dose time. The patient should make every reasonable effort to take the AZD5363 tablets(s) on time.

6.0 CRITERIA FOR SUBJECT ELIGIBILITY

Describe the characteristics of the patient/subject population.

6.1 Subject Inclusion Criteria

1. Pathologically confirmed recurrent or metastatic advanced solid tumor, for which there is no curative-intent treatment option and confirmation of the presence of AKT1, AKT2, or AKT3 mutations detected by the MSK-IMPACT assay platform or other CLIA-approved test
2. ER+ breast cancer patients must have received and progressed on Fulvestrant and be post-menopausal by the definition in 6.1.8.
3. Prostate cancer patients must have received and progressed on enzalutamide
4. Age ≥ 18 years
5. ECOG performance status ≤ 2 with no deterioration over the previous 2 weeks
6. Life expectancy of ≥ 12 weeks
7. Measurable disease as defined by the tumor specific relevant response criteria for the breast and other solid tumor cohorts (measurable disease is not required for enrollment in the prostate cancer cohort):
    a. RECIST version 1.1 criteria
    b. Prostate Cancer Clinical Trials Working Group 3 (PCWG3) criteria.
    c. RANO criteria
8. Females should be using adequate contraceptive measures (see Section 0), should not be breast feeding and must have a negative pregnancy test prior to start of dosing if of child-bearing potential or must have evidence of non-child-bearing potential by fulfilling one of the following criteria at screening:
    a. Post-menopausal defined as:
        i. Aged more than 50 years and amenorrhoeic for at least 12 months following cessation of all exogenous hormonal treatments
        ii. Estradiol, FSH and LH levels in post-menopausal range while receiving LHRH analogues for medical castration in patients with breast cancer.

   b. Documentation of irreversible surgical sterilisation by hysterectomy, bilateral oophorectomy or bilateral salpingectomy but not tubal ligation.
9. Male patients should be willing to use barrier contraception (i.e. condoms)

6.2 Subject Exclusion Criteria

1. ER+ breast cancer patients harboring the *AKT1* E17K mutation (patient population tested in MSK IRB# 14-214, study D3610C00001 part E, ClinicalTrials.gov NCT01226316).
2. Diabetes mellitus type 1
3. Fasting plasma glucose [fasting is defined as no calorific intake for at least 8 hours]:
   a. ≥ 126 mg/dL for those patients without a pre-existing diagnosis of Type 2 diabetes mellitus
   b. ≥ 167mg/dL for those patients with a pre-existing diagnosis of Type 2 diabetes mellitus
4. Glycosylated haemoglobin (HbA1C) ≥8.0%
5. Requirement for insulin for routine diabetic management and control
6. Requirement for >2 oral hypoglycaemic medications for routine diabetic management and control
7. Treatment with any of the following:
   a. Any investigational agents or study drugs from a previous clinical study within 30 days, or 5 half-lives, whichever is longer, of the first dose of study treatment
   b. Any other chemotherapy, immunotherapy or anticancer agents within 3 weeks or 5 half-lives, whichever is shorter, of the first dose of study treatment, except fulvestrant, enzalutamide or hormonal therapy with LHRH analogues for medical castration in patients with breast or prostate cancer, which are permitted
   c. Potent inhibitors or inducers or substrates of CYP3A4 or substrates of CYP2D6 within 2 weeks before the first dose of study treatment (3 weeks for St John's Wort). See section 9.5 and Appendix A.
   d. Major surgery (excluding placement of vascular access) within 4 weeks of the first dose of study treatment
   e. Radiotherapy with a wide field of radiation within 4 weeks of the first dose of study treatment
   f. Prior ATP-competitive AKT inhibitors
9. With the exception of alopecia, any unresolved toxicities from prior therapy greater than Common Terminology Criteria for Adverse Events (CTCAE) grade 1 at the time of starting study treatment
10. Spinal cord compression or brain metastases unless asymptomatic, treated and stable and not requiring steroids for at least 4 weeks prior to start of study treatment
11. As judged by the investigator, any evidence of severe or uncontrolled systemic diseases, including active bleeding diathesis, or active infection including hepatitis B, hepatitis C and human immunodeficiency virus. Screening for chronic conditions is not required.
12. Any of the following cardiac criteria:
   a. Resting corrected QT interval (QTc) > 480 msec obtained from electrocardiogram (ECG)

b. Any clinically important abnormalities in rhythm, conduction or morphology of resting electrocardiogram (ECG) eg, complete left bundle branch block, third degree heart block
c. Any factors that increase the risk of QTc prolongation or risk of arrhythmic events such as heart failure, hypokalaemia, congenital long QT syndrome, family history of long QT syndrome or unexplained sudden death under 40 years of age or any concomitant medication known to prolong the QT interval
d. Experience of any of the following procedures or conditions in the preceding 6 months and judged to be clinically significant by Principal Investigator: coronary artery bypass graft, angioplasty, vascular stent, myocardial infarction, angina pectoris, congestive heart failure New York Heart Association (NYHA) Grade ≥2
e. Uncontrolled hypotension – Systolic blood pressure (BP) <90mmHg and/or diastolic BP <50mmHg
f. Left ventricular ejection fraction (LVEF) below lower limit of normal for site.

13. Inadequate bone marrow reserve or organ function as demonstrated by any of the following laboratory values:
    a. Absolute neutrophil count < 1 x 10^9/L
    b. Platelet count < 100 x 10^9/L
    c. Haemoglobin < 9.0 g/dL
    d. ALT > 2.5 times the upper limit of normal (ULN) if no demonstrable liver metastases, or >5 times ULN in presence of liver metastases.
    e. AST > 2.5 times ULN if no demonstrable liver metastases, or >5 times ULN in presence of liver metastases.
    f. Total bilirubin > 1.5 times ULN (patients with confirmed Gilbert's syndrome may be included in the study)
    g. Creatinine >1.5 times ULN concurrent with creatinine clearance < 50 ml/min; confirmation of creatinine clearance is only required when creatinine is > 1.5 times ULN
    h. Proteinuria 3+ on dipstick analysis (See Appendix E Categorization of Quantitative Protein Urine Analysis) or >500 mg/24 hours
14. Refractory nausea and vomiting, chronic gastrointestinal diseases, inability to swallow the formulated product or previous significant bowel resection that would preclude adequate absorption of AZD5363
15. History of hypersensitivity to active or inactive excipients of AZD5363, fulvestrant and enzalutamide or drugs with a similar chemical structure or class to these agents.
16. Judgment by the investigator that the patient should not participate in the study if the patient is unlikely to comply with study procedures, restrictions and requirements

7.0 RECRUITMENT PLAN

<u>Request for Limited Waiver of Authorization:</u>

This Limited Waiver is requested to facilitate identification of study candidates with *AKT1*, *AKT2* or *AKT3* mutations. Potential research subjects will be identified by a member of the patient's treatment team, the protocol investigator, or research team here at Memorial Sloan-Kettering Cancer Center (MSKCC). Patient recruitment will occur in medical oncology clinics of the Developmental Therapeutics Program of MSKCC. If the investigator is a member of the treatment team, s/he will screen their patient's medical records for suitable research study

participants and discuss the study and their potential for enrolling in the research study. Potential subjects contacted by their treating physician will be referred to the investigator/research staff of the study.

Information Systems will provide the PI with updates (weekly or biweekly) regarding active patients in the MSKCC system that have tumors harboring *AKT1*, *AKT2* or *AKT3* mutations. However, identification of *AKT1*, *AKT2* or *AKT3* mutations is only one aspect of eligibility assessment. As such, the PI seeks to screen medical records of patients with whom he does not have a treatment relationship to identify patients with tumors harboring *AKT1*, *AKT2* or *AKT3* mutations, to further determine if these patients may be candidates for the study. The PI and coPI may screen the medical records of patients with whom they do not have a treatment relationship for the limited purpose of identifying patients who would be eligible to enroll in the study and to record appropriate contact information in order to approach these patients regarding the possibility of enrolling in the study. The PI may communicate with patient's primary medical oncologist about the possible study opportunity.

During the initial conversation between the investigator/research staff and the patient, the patient may be asked to provide certain health information that is necessary to the recruitment and enrollment process. The investigator/research staff may also review portions of the medical records at MSKCC in order to further assess eligibility. They will use the information provided by the patient and/or medical record to confirm that the patient is eligible and to contact the patient regarding study enrollment. If the patient turns out to be ineligible for the research study, the research staff will destroy all information collected on the patient during the initial conversation and medical records review, except for any information that must be maintained for screening log purposes.

In most cases, the initial contact with the prospective subject will be conducted either by the treatment team, investigator or the research staff working in consultation with the treatment team. The recruitment process outlined presents no more than minimal risk to the privacy of the patients who are screened and minimal PHI will be maintained as part of a screening log. For these reasons, we seek a (partial) limited waiver of authorization for the purposes of 1) reviewing medical records to identify potential research subjects and obtain information relevant to the enrollment process; 2) conversing with patients regarding possible enrollment; 3) handling of PHI contained within those records and provided by the potential subjects; and 4) maintaining information in a screening log of patients approached.

8.0 PRETREATMENT EVALUATION

1. Complete medical history including current medications, physical examination including evaluation of ECOG performance status, within 2 weeks prior to registration
2. Pathology review at MSKCC must confirm diagnosis of advanced solid tumor with the required mutations in *AKT1*, *AKT2* or *AKT3* as identified by the MSKCC IMPACT assay platform or other CLIA-approved test
3. The following laboratory studies will be obtained within 3 weeks prior to therapy: Complete blood count with white blood cell differential and platelet counts; Comprehensive profile (including electrolytes, bicarbonate, blood urea nitrogen (BUN), creatinine, glucose, alkaline phosphatase, aspartate aminotransferase (AST), alanine aminotransferase (ALT), total bilirubin, total protein, albumin, and glucose). Glucose level should be obtained in the fasting state (at least 8 hours). Additional baseline labs within 3 weeks prior to therapy are: PT, aPTT, magnesium, HbA1c.

4 Serum pregnancy test for women of childbearing potential within 2 weeks prior to therapy.

5 Electrocardiogram and Echocardiogram within 8 weeks prior to registration.

6 Radiologic imaging of disease with CT scan and/or MRI and Bone scan (required for the prostate cancer cohort) (required for the prostate cancer cohort) as applicable, within 4 weeks prior to registration.

7 Pretreatment Biopsy:

All patients will be asked to provide consent for collection of tumor biopsies. These should be collected from consenting patients, prior to the first dose of AZD5363.

All patients will be asked to provide consent to supply a sample of their archival tumor blocks if a sample is available. For some subjects, there may not be adequate tissue for this analysis, and this would not be considered a protocol violation.

The tumor samples will preferably be in the form of a formalin fixed paraffin embedded block (tissue derived from the diagnostic tumor or a metastatic site). If this is not possible, 10-20 slides of freshly prepared unstained 5 micron sections from the archival tumor block may be provided.

All patients must have a tumor tissue genotyped at MSKCC by the CLIA-approved Next Generation Sequencing assay (Integrated Mutational Profiling for Actionable Cancer Targets, or IMPACT) developed at MSKCC, which examines 410 genes commonly mutated in cancer using <50ng of paraffin-embedded tissue. Therefore patients must be willing to consent to IMPACT testing. However, IMPACT testing does not need to have resulted by the time of treatment on study.

Collected specimens will also undergo whole exome sequencing (WES) analyses.

Biopsies will either be done as part of standard clinical care if indicated, or alternatively as a research-non-billable (RNB) procedure if it not required as a standard of care procedure.

9.0 TREATMENT/INTERVENTION PLAN

9.1 AZD5363 administration for all tumor types other than ER+ breast and prostate cancer patients

AZD5363 will be administered as tablets, orally at the dose of 480mg, twice daily on an intermittent dosing schedule of 4 days on followed by 3 days off dosing.

Twice daily doses should be taken at approximately the same time each morning and evening approximately 12 hours apart and taken with water in a fasted state from at least 2 hours prior to the dose to at least 1 hour post-dose.

9.2 AZD5363 administration with fulvestrant therapy for ER+ breast cancer patients

AZD5363 will be administered as tablets, orally at the dose of 400mg, twice-daily, on an intermittent dosing schedule of 4 days on followed by 3 days off dosing, commencing on day 1.

Fulvestrant will be administered according to its approved dose of 500 mg intramuscularly on days 1, 15, 29(or Cycle 2 Day 1) and then every 4 weeks with a window of +/- 3 days.

9.3 AZD5363 administration with enzalutamide therapy for prostate cancer patients

AZD5363 will be administered as tablets, orally at the dose of 400mg, twice-daily, on an intermittent dosing schedule of 4 days on followed by 3 days off dosing, commencing on day 1.

Enzalutamide will be administered according to its approved dose of 160 mg PO once daily

Enzalutamide can be taken at any time during the day, but should be taken at the same time consistently

9.4 Restrictions

The following restrictions apply while the patient is receiving study treatment and for the specified times before and after:

Females of child-bearing potential should use two forms of highly reliable methods of contraception from the time of screening until 4 weeks after discontinuing study treatment. Acceptable methods of contraception include:

- Established use of oral, injected or implanted hormonal methods of contraception.
- Placement of an intrauterine device (IUD) or intrauterine system (IUS).
- Barrier methods of contraception: Condom or Occlusive cap (diaphragm or cervical/vault caps) with spermicidal foam/gel/film/cream/suppository.
- Male sterilisation (with the appropriate post-vasectomy documentation of the absence of sperm in the ejaculate).
- True abstinence.

It is not known whether AZD5363 has the capacity to affect the metabolism of hormonal contraceptives, so hormonal contraception should also be combined with a barrier method of contraception

Male patients should use barrier contraception (ie, condoms) for 16 weeks after discontinuation of study drug. It is not known whether the preclinical changes seen in the male animal reproductive organs, after treatment with AZD5363, will be fully reversible or will permanently affect the ability to produce healthy sperm following treatment. Therefore, if male patients wish to father children they should be advised to arrange for freezing of sperm samples prior to the start of study treatment.

9.5 Concomitant treatments

AZD5363:

Information on any treatment in the 4 weeks prior to starting study treatment and all concomitant treatments given during the study, with reasons for the treatment, will be recorded in the Case Report Form (CRF). If medically feasible patients taking regular medication, with the exception of potent inhibitors or inducers or substrates of CYP3A4 or substrates of CYP2D6 (see Section 6.2 exclusion and Appendix A)) should be maintained on it throughout the study period.

Other anticancer agents (with the exception of fulvestrant, enzalutamide or hormonal therapy with LHRH analogues for medical castration in patients with prostate cancer and for menopause induction in pre-menopausal patients with breast cancer), investigational agents and radiotherapy should not be given while the patient is on study treatment although radiation for palliation at focal sites is permitted.

Blood transfusions are allowed at any time during the study.

Granulocyte colony stimulating may be considered.

Patients may receive treatment with bisphosphonates or RANKL inhibitors for the treatment of bone metastases.

Patients may take warfarin or a coumarin preparation but it is recommended that they should have their anticoagulation monitored carefully and dose adjusted accordingly.

Patients may take corticosteroids, however, increased vigilance is recommended on electrolyte and/or glucose levels due to the potential for corticosteroid-related metabolic disturbance.

Supportive care and other medications that are considered necessary for the patient's well-being, may be given at the discretion of the investigator.

A list of Concomitant Treatment Cautions and Restrictions for AZD5363 are provided in Appendix A

Enzalutamide:
Avoid strong CYP2C8 inhibitors, as they can increase the plasma exposure to enzalutamide. If co-administration is necessary, reduce the dose of enzalutamide.

Avoid strong CYP3A4 inducers as they can decrease the plasma exposure to enzalutamide. If co-administration is necessary, increase the dose of enzalutamide.

Avoid CYP3A4, CYP2C9, and CYP2C19 substrates with a narrow therapeutic index, as enzalutamide may decrease the plasma exposures of these drugs. If enzalutamide is co-administered with warfarin (CYP2C9 substrate), conduct additional INR monitoring.

See section **11.2.3** for guidance on dose modifications with concomitant use of strong CYP2C8 inhibitors and strong CYP3A4 inducers

9.6 General Considerations

All and any treatment and/or clinic visits may occur 3 days before or after the scheduled date, if necessary for medical or logistical reasons. Treatment/visit delay of up to 21 days may be allowed for medical or logistical reasons. Reasons for such changes in schedule will be documented in the medical record.

Guidelines regarding dose delay or dose reduction are provided in Section 11.

Patients will be provided with a pill diary at the start of each cycle and will be required to bring completed pill diary to clinic for the start of each new cycle for the first 24 weeks and then every 2 cycles thereafter.

Treatment may be discontinued at any time for progression of disease, unacceptable side effects, life-threatening toxicity, patient request, or investigator judgment.

9.7 Use of Metformin

Metformin is recommended for the management of potential AZD5363-induced hyperglycemia. Investigators should exercise caution in the dosing and management of patients receiving the metformin plus AZD5363 combination and must be vigilant for signs of renal impairment and metformin toxicity, such as lactic acidosis and hypoglycemia, namely: lethargy, hypotension, poor urine output, drowsiness, irritation, tachypnea, sweating, diarrhea, and vomiting.

Metformin should only be given on the days when AZD5363 is also given (the half-life of AZD5363 is approximately 8 to 15 hours), and should be withdrawn when treatment with AZD5363 is also withdrawn, unless otherwise clinically indicated.

9.8 Study procedures for all patients

On Day 1 of each Cycle, patients begin oral AZD5363 as detailed in section 9.1-9.3 above.

Research bloods for cfDNA analysis will be drawn at the time of every study visit (for details, see Section 10.2).

Radiologic imaging will be performed after every 2 cycles (8 weeks) of therapy for the first 24 weeks of therapy, after which they will occur every 12 weeks. Radiologic imaging (typically, either CT scans and/or MRI scans and Bone scans-required for the prostate cancer cohort) will be performed, with a +/- 7 day window.

Clinic visits with laboratory studies are required at the start of each cycle.

10.0 EVALUATION DURING TREATMENT/INTERVENTION

Patients will be examined on Day 1(+/-3 days) of each cycle for the first 24 weeks and then every 2 cycles (approximately every 8 weeks) thereafter, with additional clinic visits scheduled as needed.

Radiologic imaging will be performed after every 2 cycles (8 weeks) of therapy for the first 24 weeks and then every 12 weeks thereafter. Radiologic imaging (typically, either CT scans and/or MRI scans and Bone scans-required for the prostate cancer cohort) will be performed to evaluate the disease status. Additional imaging may be obtained as clinically indicated. For patients who develop intercurrent medical problems or logistical issues, the timing of radiologic imaging may be altered at the discretion of the treating physician. Reasons for alterations regarding the timing of scans will be provided in the medical record. There is a +/- 7 day window for all scans.

10.1 Study plan

	Screening[a]	Day 1 of each Cycle (±3 days)	End of treatment	28 day follow-up[l] (±7 days)
AZD5363 dispensed[b]		Taken 4 days on/3 days off		
Enzalutamide dosing[b] (Prostate cancer only)		Taken continuously daily		
Fulvestrant dosing[b] (ER+ve breast only)		X (and day 15 during cycle 1)		
History/Physical[c]	X	X	X	
Weight and full vitals check[c]	X	X	X	
Complete blood cell counts (CBC)	X	X	X	
Comprehensive Metabolic Panel and Direct Bilirubin[d]	X	X	X	
Magnesium	X			
Tumor markers[e]		X		
Urinalysis [f]	X	X		
PT, aPTT	X		X	
HbA1c & Lipid Panel[g]	X	X	X	
Echocardiogram	X			
EKG [h]	X	X[h]	X	
Pregnancy Test[i]	X			
Imaging Studies[j]	X	X	X	
Research blood(cfDNA)[k]	X	X	X	
Pre-treatment tumor biopsy (optional) or provision of archival tumor blocks if available	X			
Tumor biopsy on progression (optional)				X
Conconmittant medications	X	X	X	X
Adverse Event Monitoring	Continuous			X

a. See Section 8 for required time frames for pre-treatment studies
b. AZD5363 480mg bid 4 days on/3 days off. If an ER+ breast cancer patient - Fulvestrant therapy will be administered on D1,15,29 (or cycle 2 day 1) and then every 4 weeks and AZD5363 will be dosed at 400mg bid 4 days on/3 days off. If a Prostate cancer patient- Enzalutamide 160mg will be taken daily orally and AZD5363 will be dosed at 400mg bid 4 days on/3 days off.

c. Patients will be evaluated by a physician and/or nurse at the start of every cycle for the first 24 weeks and then every 2 cycles (approximately every 8 weeks) thereafter, with additional clinic visits scheduled as needed. Full vitals will be done at these visits, including blood pressure, heart rate, respiratory rate, and body temperature.
d. Fasting for screening, Day 1 of Cycle 1 and 2. Thereafter, fasting gluocse will be repeated if non-fasting glucose is >180 mg/dL.
e. As applicable to disease type.
f. If 3+ proteinuria is identified by dipstick assessment, a 24-hour urine collection for formal quantification of the level of protein excretion should be performed.
g. HbA1c and fasting lipids at screening and then every 12 weeks (starting at Cycle 4).
h. 12-lead surface EKG will be obtained Screening and Day 1 of Cycles 1 and 2, and then as clinically indicated thereafter.
i. Serum pregnancy test for women of childbearing potential within 14 days prior to therapy.
j. Radiologic imaging (CT scan and/or MRI and Bone scan-required for the prostate cancer cohort) of sites of disease will be obtained at baseline and after every 2 cycles (8 weeks) to assess for response to treatment. After 24 weeks of study drug treatment, radiologic imaging may be performed after every 3 cycles (12 weeks). Patients who discontinue for reasons other than progression should be imaged when they come off study and then every 3 months until progression of disease or beginning of alternative anticancer therapy.There is a +/- 7 day window for all scans.
k. cfDNA research blood will be collected at each study visit.
l. The 28 day follow-up and off study date will be approximately 28 days (+/- 7 days) from the end of treatment date.

10.2 Collection of plasma for analysis of cfDNA

All patients will be requested to provide plasma samples for the extraction and analysis of circulating tumor DNA (cfDNA). The cfDNA will be used for ddPCR and NGS analysis.

All patients will be required to provide:

2x 10 mL blood sample for preparation of plasma at every study visit.

Time points for collection of cfDNA samples are presented in section 10.1(study plan). Residual material may be used for future exploratory biomarker research.

10.3 Off Study Assessment

A follow up assessment of adverse events and concomitant medications will be scheduled approximately 28 days (± 7 days) from date that the patient is withdrawn from study. The end of treatment day will be the final day of study treatment. The off study date will be 28 days (+/- 7 days) thereafter.

The following assessments will be conducted during after the end of treatment:

a. Optional Post-Progression research tumor biopsy for repeat sequencing (NGS & WES) to determine mechanism(s) of acquired resistance.
b. Patients who discontinue for reasons other than progression should be imaged when they come off study and then every 3 months until progression of disease or beginning of alternative anticancer therapy.

11.0 TOXICITIES/SIDE EFFECTS

11.1 Toxicity Management: general considerations

The following subsections provide guidelines for dose delays and/or dose reductions for treatment-related toxicities in relation to AZD5363, unless otherwise specified.

Identified toxicities of AZD5363 are:

Constitutional: Hypersensitivity
Dermatologic: Rash,
Endocrine: Hyperglycemia
Gastrointestinal: Diarrhea,

Identified toxicities of fulvestrant are:

The most common adverse reactions occurring in ≥5% of patients receiving 500 mg FASLODEX were: injection site pain, nausea, bone pain, arthralgia, headache, back pain, fatigue, pain in extremity, hot flash, vomiting, anorexia, asthenia, musculoskeletal pain, cough, dyspnea, and constipation

Increased hepatic enzymes (ALT, AST, ALP) occurred in >15% of FASLODEX users and were not dose-dependent

Because Fulvestrant is administered intramuscularly, it should be given with caution in patients with bleeding diathesis, thrombocytopenia, or anticoagulant use. For further details regarding the safety profile of fulvestrant, refer to the fulvestrant package insert or SmPC.

Identified toxicities of enzalutamide are:

The most common adverse reactions (≥ 10%) that occurred more commonly (≥ 2% over placebo) in the enzalutamide-treated patients from the two randomized clinical trials were asthenia/fatigue, back pain, decreased appetite, constipation, arthralgia, diarrhea, hot flush, upper respiratory tract infection, peripheral edema, dyspnea, musculoskeletal pain, weight decreased, headache, hypertension, and dizziness/vertigo.

Seizure occurred in 0.9% of patients receiving enzalutamide who previously received docetaxel, and in 0.1% of patients who were chemo-naive. Permanently discontinue enzalutamide in patients who develop a seizure during treatment.

Posterior Reversible Encephalopathy Syndrome (PRES) In post approval use, there have been reports of PRES in patients receiving enzalutamide. PRES is a neurological disorder which can present with rapidly evolving symptoms including seizure, headache, lethargy, confusion, blindness, and other visual and neurological disturbances, with or without associated hypertension. A diagnosis of PRES requires confirmation by brain imaging, preferably MRI. Discontinue enzalutamide in patients who develop PRES. For further details regarding the safety profile of enzalutamide, refer to the enzalutamide package insert or SmPC.

In the event of an AE which the investigator considers to be related to the administration of study treatment, supportive therapy should be given at the discretion of the investigator.

11.2 Toxicity dose modifications

11.2.1 Toxicity dose modifications for AZD5363

If a patient experiences a clinically significant and/or unacceptable toxicity considered to be related to AZD5363, AZD5363 dosing will be interrupted or the dose reduced and supportive therapy administered as required.

Patients should receive dose reductions of AZD5363 in 80 mg decrements and should have no more than 2 dose reductions. Patients who have had one or two dose reductions and who have demonstrated an acceptable response to the dose interruption may restart at their prior dose, at the discretion of the Investigator. Repeat dose reductions beyond the 2nd reduction should first be discussed with the PI or co-PI.

Table 3 - AZD5363 dose reductions

AZD5363 starting dose	1st dose reduction	2nd dose reduction
480mg bid	400mg bid	320mg bid
400mg bid	320mg bid	240mg bid (this will change to 200mg as the new drug formulation will not be produced in 80mg tablets)

All dose modifications and interruptions (including any missed doses due to AEs), and the reasons for the dose modifications/interruptions are to be recorded in the CRF.

11.2.2 Toxicity dose modifications for fulvestrant

A 250-mg dose is recommended in patients with moderate hepatic impairment (Child-Pugh class B).

11.2.3 Toxicity dose modifications for enzalutamide

If a patient experiences a ≥ Grade 3 toxicity or an intolerable side effect, withhold dosing for 1 week or until symptoms improve to ≤ Grade 2, then resume at the same or a reduced dose (120 mg or 80 mg), if warranted.

The concomitant use of strong CYP2C8 inhibitors should be avoided if possible. If patients must be co-administered a strong CYP2C8 inhibitor, reduce the enzalutamide dose to 80 mg once daily. If co-administration of the strong inhibitor is discontinued, the enzalutamide dose should be returned to the dose used prior to initiation of the strong CYP2C8 inhibitor.

The concomitant use of strong CYP3A4 inducers should be avoided if possible. If patients must be co-administered a strong CYP3A4 inducer, increase the enzalutamide dose from 160 mg to 240 mg once daily. If co-administration of the strong CYP3A4 inducer is discontinued, the

enzalutamide dose should be returned to the dose used prior to initiation of the strong CYP3A4 inducer.

11.3 Gastro-intestinal toxicity related to AZD5363

For diarrhea that is clinically significant or intolerable and causally related to treatment with AZD5363, institute appropriate anti-diarrheal treatment with loperamide or similar (Appendix F Patient instructions for the management of Diarrhea). If clinically appropriate or if toxicity does not improve or remains clinically intolerable, despite optimal treatment, withhold AZD5363 and consider dose reduction as in 11.2.1.

11.4 Glucose abnormalities related to AZD5363

Specific management of the hyperglycemia will be according to local practice; however, the principles in the blood glucose intervention plan (see Figure 3) should be followed.

Figure 3 **Blood glucose intervention plan**

Grade 0 (FBG<125 mg/dL)

- Educate patient on risk of hyperglycemia and review signs and symptoms of hyperglycemia

Grade 1 (FBG>125-160 mg/dL)

- Continue study drug
- Therapeuic lifestyle changes
- Consider home glucose monitoring

Grade 2 (FBG>160-250mg/dL)

- Continue study drug unless symptomatic
- Home glucose monitoring twice weekly[1]
- Start metformin extended release 500 mg once daily , titrate to max dose[2]
- Consider switching to or adding second agent DPP-4 inhibitor, Sulfonylureas or TZDs when metformin at max dose or poorly tolerated
- Consider insulin therapy for patients who do not tolerate PO regimen
- Arrange endocrine consult once hyperglycemic agent initiated

Grade 3 (FBG>250-500 mg/dL)

- Hold study drug
- Daily home glucose monitoring- alternating fasting and non-fasting
- Place endocrine consult
- Consider start basal insulin
- Restart study drug when FGB<250 mg/dL and patient has no symptoms

Grade 4 (FBG>500 mg/dL)

- Hold study drug
- BID home glucose monitoring
- Endocrine consult
- Refer to UCC for symptomatic management (could avoid with endo input)

1: Daily blood glucose monitoring for patients on insulin or sulfonylureas

2: Should only be given on days receiving study drug

11.5 Hepatotoxicity

AZD5363

Following discharge from the clinic, any patients experiencing symptoms consistent with acute liver dysfunction such as unexplained pruritus, jaundice or right upper quadrant pain will be advised to temporarily stop study treatment and promptly contact the clinic for clinical assessment and liver biochemistry testing. Investigation and management of these patients and any patients with AST or ALT results > 8 x ULN identified at any time during the study will be according to local practice, however, the principles of the FDA Draft Guidance for evaluation of Drug-Induced Liver Injury should be followed. If a patient exhibits an AST, ALT result in excess of 10 x ULN, or AST or ALT in excess of 8 x ULN in combination with a doubling of bilirubin from baseline, which is considered to be related to study drug, they will not be permitted to restart study treatment.

Fulvestrant

Fulvestrant is metabolized primarily in the liver. A 250-mg dose is recommended in patients with moderate hepatic impairment (Child-Pugh class B). FASLODEX has not been evaluated in patients with severe hepatic impairment (Child-Pugh class C)

11.6 Skin toxicity related to AZD5363

If a patient experiences a maculo-papular rash, guidance provided below should be followed.

Grade 1 or 2: Maintain dose level. Consider skin toxicity therapy*

Intolerable Grade 2 or ≥ Grade 3: Hold AZD5363 treatment until resolution ≤ Grade 2. The need for oral or topical treatments* can be at the discretion of the investigator and may be accompanied by a dermatology consult. When toxicity resolves to Grade 2 or better, dose reductions may be at the discretion of the investigator.

*Options include: topical steroids moderate strength bid, oral antihistamine, oral steroids

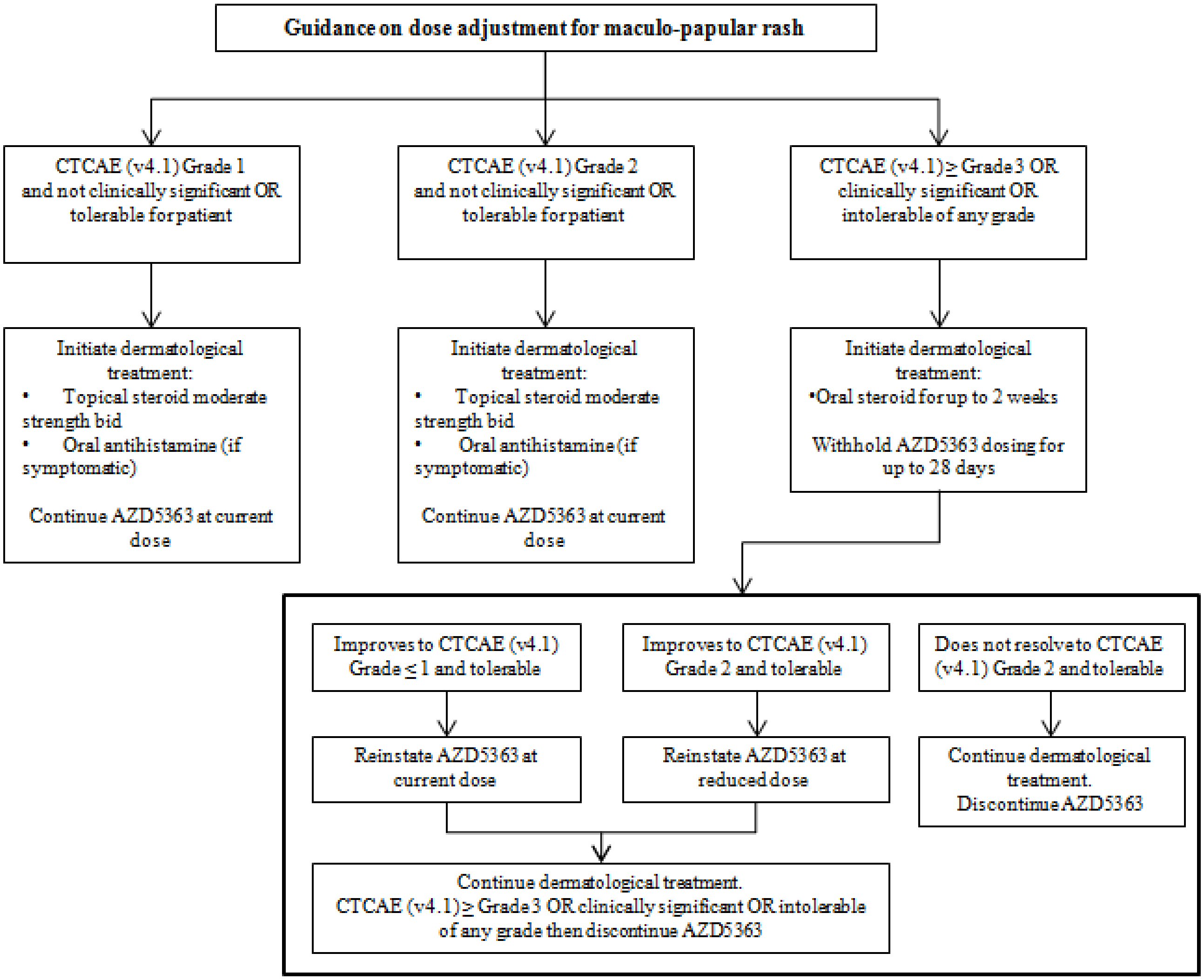

12.0 CRITERIA FOR THERAPEUTIC RESPONSE/OUTCOME ASSESSMENT

Objective response rate is the primary endpoint of this trial and will be assessed by standard criteria. For the purposes of this study, patients should be re-evaluated every 8 weeks for the first 24 weeks and then every 12 weeks thereafter.

Response and progression will be evaluated in this study according to RECIST v1.1 (see Appendix B), PCWG3 (See Appendix C), RANO (See Appendix D) and prostate-specific antigen (PSA) measurement, as appropriate to primary tumor type.

12.1. Definitions

Evaluable for toxicity. All patients will be evaluable for toxicity from the time of their first treatment with AZD5363, AZD5363+ fulvestrant, or AZD5363+ enzalutamide, as applicable.

Evaluable for objective response.

Only patients who have received at least one dose of AZD5363 and have had their disease re-evaluated will be considered evaluable for response. These patients will have their response classified according to the definitions stated in appendix above or by PSA measurement (in prostate cancer patients without visceral and/or nodal disease at baseline)

A response is defined as any of the following: a response according to RECIST v1.1 (sum of partial responses (PRs) and complete responses (CRs) in patients with measurable visceral and/or nodal disease at baseline); PCWG3; RANO as applicable **or** a reduction in the PSA level of 50% or more (in prostate cancer patients without visceral and/or nodal disease at baseline), with a confirmatory assessment at least 4 weeks later.

13.0 CRITERIA FOR REMOVAL FROM STUDY

If at any time the patient develops progressive disease he/she will be taken off study and referred for alternative therapy.

Patients may be removed from the study for protocol non-compliance.

If at any time the patient develops unacceptable toxicity he/she will be removed from study.

Participants can be removed from the study at any time if the study doctor feels that it is in their best interest to do so.

14.0 BIOSTATISTICS

14.1 General Considerations

This will be an open label, single institution, non-randomized, pilot study for patients with advanced solid tumors harboring mutations in AKT1, AKT2, or AKT3, to evaluate the anti-tumor efficacy of AZD5363 in this population. The target accrual will be 12 patients in each cohort for a total of 36 patients for 3 cohorts: 2 disease specific cohorts and one other tumor cohort. Each cohort will be analyzed separately. If observed responses are confined to AKT1 E17K mutant patients only, further study of non- E17K AKT1, or any AKT2, AKT3 mutant tumors would not be pursued. Accrual rate is expected to be 1 per month and the study is expected to complete within 3 years. Analyses for all the objectives (primary, secondary and exploratory) will be performed separately in each cohort.

14.2 Primary Endpoint

Response Rate:

Primary analysis:

The primary endpoint is ORR defined as the proportion of patients with a confirmed response of CR or PR using either modified RECIST Version 1.1 (if measurable visceral

and/or nodal disease is present) or ≥ 50% PSA decrease from baseline (in prostate cancer patients without visceral and/or nodal disease at baseline), measured as a binary covariate: responder yes, no and will be analyzed assuming binomial proportions. With 12 patients treated in each cohort, this study aims to obtain preliminary evidence of antitumor activity in each cohort. If the drug is efficacious, we expect to see at least 3 responders out of 12 patients in each cohort. Zero, 1, or 2 responses out of 12 patients will be considered non promising. Given that this protocol investigates a targeted therapy and not a standard chemotherapy regimen in a patient population with rare genotypes, a RR of >=3/12 is considered clinically meaningful activity in this setting.

Further analyses of the genomic profiles of the enrolled patients will attempt to investigate if there are any differential patterns between responders and non-responders (see correlative studies below).

The table below provides the probability of observing 3 or more responders out of 12 patients under various true hypothetical rates of response:

hypothetical rate of response:	Probability of seeing <=2 response out of 12 pts	Probability of seeing >= 3 responses out of 12 pts
0.05	0.98	0.02
0.15	0.74	0.26
0.25	0.39	0.61
0.30	0.25	0.75
0.35	0.15	0.85

<u>Secondary analysis:</u>

At the end of the study a heterogeneity test will be used to determine if response rates are different across the three cohorts using a Chi square or Fisher's exact test as appropriate. All tests will be two-sided and at the 0.05 alpha level. If the study fails to reject the hypothesis that RR are the same across cohorts, given the small sample size and the lack of power, these results will not preclude the scenario that the RR /efficacy in different cohorts are potentially different.

14.3 Secondary Endpoints

Safety:

- Toxicities will be assessed according to NCI common toxicity criteria (CTC) version 4.0. No safety stopping rules will be implemented, as the safety profile of these agents and the MTD have been established in prior and ongoing studies (see section 3.3)

Efficacy:

- Clinical benefit rate (CBR) (complete response, partial response, or stable disease) at 24 weeks. This will be analyzed assuming binomial proportions.
- Progression-free survival (PFS) defined from start of treatment to progression or death whichever occurs first. Patients will be censored at last follow up or at the initiation of subsequent therapy if they have not progressed. The Kaplan Meier method will be used to estimate median PFS.
- Additional objectives for Prostate cohort:
    - Estimate the proportion of patients without PSA progression by PCWG3 criteria at 6 months. PSA progression will be analyzed as a time to event variable using the Kaplan Meier method.
    - Determine the 12 week PSA. RR defined as the percentage of change in PSA from baseline to 12 weeks or earlier for those who discontinue therapy, as well as the maximum decline in PSA that occurs at any point after treatment reported for each patient will be presented using a waterfall plot. The Kaplan Meier method will be used to estimate PFS for the prostate cohort
    - Evaluate the PSA-PFS by PCWG3 criteria
        - Definition of PSA progression by PCWG3 criteria (Appendix C Table 4):
            - In patients in whom there is a decline in PSA from baseline: PSA progression is defined from start of treatment to first PSA increase that is ≥25% and ≥2 ng/mL above the nadir, and which is confirmed by a second value 3 or more weeks later (i.e., a confirmed rising trend)
            - In patients in whom there no decline in PSA from baseline: PSA progression is defined as PSA increase that is ≥25% and ≥2 ng/mL from baseline after 12 weeks.

14.4 Correlative/Exploratory objectives

- Examine the clonality and genetic configuration of the *AKT* sensitizing mutation in the pre-treatment specimens of study patients and their associated clinical response to AZD5363.
    - We will establish a 2-tiered clonality status (Clonal, or subclonal) and genetic configuration status (Allelic imbalance, or Allelic balance) of the sensitizing *AKT* mutation in each patient and compare this classification to the best overall response to AZD5363 (binary outcome) in the corresponding patient, using a two-sided Chi-Square or Fisher's exact test and a Type I error of 5%. A definitive power calculation is not feasible due to lack of historical estimates of the clonality status of *each AKT* mutation and treatment effect in this population. Moreover, due to the limited sample size, these analyses are necessarily exploratory and hypothesis generated by nature.
- Examine the pattern of co-mutated genes in *AKT*-mutant tumors and their association with treatment response or resistance
    - At the patient level, associations of pretreatment pattern of co-mutated genes (grouped by pathway for e.g. mutations affecting the PI3K-Akt-mTOR pathway – binary covariate) and response (best overall response; binary outcome covariate defining responders and non-responders) will be assessed. Association between response and presence of co-mutated genes will be assessed with Fisher's exact test.

- Describe possible mechanisms of acquired resistance to AKT inhibition.
    - Sequencing data from pre- and post-treatment specimens of patients that initially responded to AZD5363 will be compared to identify newly acquired mutations or DNA copy number alterations. Serial cfDNA samples will also be evaluated for emerging mutations using MSK-IMPACT.
    - This will be assessed descriptively. Given that the number of patients and recurrence rate of specific events will be low, this analysis will be primarily descriptive with graphical representation in order to uncover any trends such as a pathway specific clustering of resistance mutations or second-site mutations or focal amplifications that might inform potential mechanisms of acquired resistance.
    - Results will be used to generate future hypotheses that will be tested in larger patient samples.

15.0 RESEARCH PARTICIPANT REGISTRATION AND RANDOMIZATION PROCEDURES

15.1 Research Participant Registration

Confirm eligibility as defined in the section entitled Criteria for Subject Eligibility. Obtain informed consent, by following procedures defined in section entitled Informed Consent Procedures. During the registration process registering individuals will be required to complete a protocol specific Eligibility Checklist. The individual signing the Eligibility Checklist is confirming whether or not the participant is eligible to enroll in the study. Study staff are responsible for ensuring that all institutional requirements necessary to enroll a participant to the study have been completed. See related Clinical Research Policy and Procedure #401 (Protocol Participant Registration).

15.2 Randomization

There is no randomization in this open label study

16.0 DATA MANAGEMENT ISSUES

A Clinical Research Associate (CRA) and/or a Clinical Research Coordinator (CRC) will be assigned to the study. The responsibilities of the CRA/CRC include protocol compliance, data collection, abstraction and entry, data reporting, regulatory monitoring, problem resolution and prioritization, and coordinate the activities of the protocol study team.

The data collected for this study will be entered into a secure database. Source documentation will be available to support the computerized patient record.

16.1 Quality Assurance

Monthly registration reports will be generated to monitor patient accruals and completeness of registration data. Routine data quality reports will be generated to assess missing data and inconsistencies. Accrual rates and extent and accuracy of evaluations and follow-up will

be monitored periodically throughout the study period and potential problems will be brought to the attention of the study team for discussion and action.

16.2 Data and Safety Monitoring

The Data and Safety Monitoring (DSM) Plans at Memorial Sloan-Kettering Cancer Center were approved by the National Cancer Institute in September 2001. The plans address the new policies set forth by the NCI in the document entitled "Policy of the National Cancer Institute for Data and Safety Monitoring of Clinical Trials" which can be found at: http://cancertrials.nci.nih.gov/researchers/dsm/index.html. The DSM Plans at MSKCC were established and are monitored by the Office of Clinical Research. The MSKCC Data and Safety Monitoring Plans can be found on the MSKCC Intranet at: *http://mskweb5.mskcc.org/intranet/_assets/_tables/content/359709/DSMPlans07.pdf*

There are several different mechanisms by which clinical trials are monitored for data, safety and quality. There are institutional processes in place for quality assurance (e.g., protocol monitoring, compliance and data verification audits, therapeutic response, and staff education on clinical research QA) and departmental procedures for quality control, plus there are two institutional committees that are responsible for monitoring the activities of our clinical trials programs. The committees: *Data and Safety Monitoring Committee (DSMC)* for Phase I and II clinical trials, and the *Data and Safety Monitoring Board (DSMB)* for Phase III clinical trials, report to the Center's Research Council and Institutional Review Board.

During the protocol development and review process, each protocol is assessed for its level of risk and degree of monitoring required. Every type of protocol (e.g., NIH sponsored, in-house sponsored, industrial sponsored, NCI cooperative group, etc.) will be addressed and the monitoring procedures will be established at the time of protocol activation.

17.0 PROTECTION OF HUMAN SUBJECTS

Potential risks to human subjects include drug related toxicity, pain and discomfort associated with AZD5363, fulvestrant and enzalutamide side effects (Section 11), , phlebotomy, and possible psychological discomfort from the stresses associated with obtaining imaging studies (eg, CT scan, Bone scan). All efforts will be made to avoid any complication by completely reviewing patients' symptoms, providing appropriate management, and monitoring blood tests.

If an adverse medical event occurs, the patient should first contact the primary oncologist or the Principal Investigator. At nights and on weekends, there is an oncology physician on call at all times. Patients may either call or come directly to the urgent care center at Memorial Hospital (or to their local emergency room) to be seen. Patients suffering serious adverse reactions must be carefully followed and all follow-up information also recorded.

Participation in this trial is voluntary. Depending on the specific details of the situation, patient options without being in a study might include:

- Other palliative chemotherapy off study.
- Participation in a different clinical trial
- Best supportive care

The patient will be responsible for all costs related to treatment and complications of treatment. Costs to the patient (third party insurer) will include the costs of fulvestrant (if an ER+ breast cancer patient), enzalutamide (if prostate cancer patient) hospitalizations, routine blood tests and diagnostic studies, office visits, baseline EKG and doctor's fees. Patients will not be charged for any research biopsies or tests performed on research specimens (cfDNA analysis, tumor tissue genotyping analysis with MSKCC IMPACT Assay)

Inclusion of Children in Research

This protocol/project does not include children because the number of children is limited and because the majority are already accessed by a nationwide pediatric cancer research network. This statement is based on exclusion 4b of the NIH Policy and Guidelines on the Inclusion of Children as Participants in Research Involving Human Subjects.

17.1 Privacy

MSKCC's Privacy Office may allow the use and disclosure of protected health information pursuant to a completed and signed Research Authorization form. The use and disclosure of protected health information will be limited to the individuals described in the Research Authorization form. A Research Authorization form must be completed by the Principal Investigator and approved by the IRB and Privacy Board (IRB/PB).

17.2 Serious Adverse Event (SAE) Reporting

An adverse event is considered serious if it results in ANY of the following outcomes:

- Death
- A life-threatening adverse event
- An adverse event that results in inpatient hospitalization or prolongation of existing hospitalization
- A persistent or significant incapacity or substantial disruption of the ability to conduct normal life functions
- A congenital anomaly/birth defect
- Important Medical Events (IME) that may not result in death, be life threatening, or require hospitalization may be considered serious when, based upon medical judgment, they may jeopardize the patient or subject and may require medical or surgical intervention to prevent one of the outcomes listed in this definition

Note: Hospital admission for a planned procedure/disease treatment is not considered an SAE.

SAE reporting is required as soon as the participant starts investigational treatment/intervention. SAE reporting is required for 30-days after the participant's last investigational treatment/intervention. Any event that occur after the 30-day period that is unexpected and at least possibly related to protocol treatment must be reported.
Please note: Any SAE that occurs prior to the start of investigational treatment/intervention and is related to a screening test or procedure (i.e., a screening biopsy) must be reported.

All SAEs must be submitted in PIMS. If an SAE requires submission to the HRPP office per IRB SOP RR-408 'Reporting of Serious Adverse Events', the SAE report must be submitted within 5 calendar days of the event. All other SAEs must be submitted within 30 calendar days of the event.
The report should contain the following information:
• The date the adverse event occurred
• The adverse event
• The grade of the event
• Relationship of the adverse event to the treatment(s)
• If the AE was expected
• Detailed text that includes the following
o An explanation of how the AE was handled
o A description of the participant's condition
o Indication if the participant remains on the study
• If an amendment will need to be made to the protocol and/or consent form
• If the SAE is an Unanticipated Problem

For IND/IDE protocols:
The SAE report should be completed as per above instructions. If appropriate, the report will be forwarded to the FDA by the IND Office

17.2.1 SAE Reporting to AstraZeneca

Every SAE, occurring after the patient has been registered to the study and until 4 weeks after the patient has stopped study treatment/participation must be reported to AstraZeneca within 24 hours (excluding weekends and holidays) of learning of its occurrence. Any SAEs experienced after this 4-week period should only be reported to AstraZeneca if the investigator suspects a causal relationship to the study drug. Recurrent episodes, complications, or progression of the initial SAE must be reported as follow-up to the original episode within 24 hours (excluding weekends and holiday) of the investigator receiving the follow-up information. An SAE occurring at a different time interval or otherwise considered completely unrelated to a previously reported one should be reported separately as a new event.

Unblinded Suspected Unexpected Serious Adverse Reactions (SUSARs) should be reported as individual case reports as they occur and in parallel to reporting to the regulatory authority.

Blinded Serious Adverse Events (SAEs) and Suspected Serious Adverse Reactions (SSAR's) should be reported as individual case reports as they occur.

The investigator must assess and record the relationship of each SAE to AZD5363, complete the SAE Report in English, and send the completed, signed form to the AstraZeneca Product Safety mailbox: AEMailboxClinicalTrialTCS@astrazeneca.com or by fax to +1-866-984-7229 within 24 hours. The original copy of the SAE report and the fax confirmation sheet must be kept within the Trial Master File at the study site.

Follow-up information is sent to the same person to whom the original SAE Report Form was sent, using a new SAE Report Form stating that this is a follow-up to the previously reported SAE and giving the date of the original report. Each re-occurrence, complication, or progression of the original event should be reported as a follow-up to that event regardless of when it occurs. The follow-up information should describe whether the event has resolved or continues, if and how it was treated, whether the blind was broken or not (if applicable), and whether the patient continued or withdrew from study participation.

The principal investigator has the obligation to report all serious adverse events to the FDA, IRB, and AstraZeneca). All correspondence with the FDA will go through MSKCC's IND office.

For Comparator Drugs/Secondary Suspects (Concomitant Medications), all serious adverse experiences will be forwarded to the product manufacturer by the investigator.

The SAE will also be reported to the FDA through the IND Office and that the report must include the FDA assigned BB-IDE, BB-IND or IND number and name.

17.2.1.1 Adverse Event Definition

The study will be performed in accordance with ethical principles that have their origin in the Declaration of Helsinki and are consistent with ICH/Good Clinical Practice, and applicable regulatory requirements Subject data protection.

The International Conference on Harmonization (ICH) Guideline for Good Clinical Practice (GCP) E6(R1) defines an AE as:
Any untoward medical occurrence in a patient or clinical investigation subject administered a pharmaceutical product and which does not necessarily have a causal relationship with this treatment. An AE can therefore be any unfavorable and unintended sign (including an abnormal laboratory finding), symptom, or disease temporally associated with the use of a medicinal product, whether or not considered related to the medicinal product.

An AE includes but is not limited to any clinically significant worsening of a subject's pre-existing condition. An abnormal laboratory finding (including ECG finding) that requires an action or intervention by the investigator, or a finding judged by the investigator to represent a change beyond the range of normal physiologic fluctuation, should be reported as an AE.

Adverse events may be treatment emergent (i.e., occurring after initial receipt of investigational product) or nontreatment emergent. A nontreatment-emergent AE is any new sign or symptom, disease, or other untoward medical event that begins after written informed consent has been obtained but before the subject has received investigational product.

Elective treatment or surgery or preplanned treatment or surgery (that was scheduled prior to the subject being enrolled into the study) for a documented pre-existing condition, that did not worsen from baseline, is not considered an AE (serious or nonserious). An untoward medical event occurring during the prescheduled elective procedure or routinely scheduled treatment should be recorded as an AE or SAE.

The term AE is used to include both serious and non-serious AE's.

18.0 INFORMED CONSENT PROCEDURES

Before protocol-specified procedures are carried out, consenting professionals will explain full details of the protocol and study procedures as well as the risks involved to participants prior to their inclusion in the study. Participants will also be informed that they are free to withdraw from the study at any time. All participants must sign an IRB/PB-approved consent form indicating their consent to participate. This consent form meets the requirements of the Code of Federal Regulations and the Institutional Review Board/Privacy Board of this Center. The consent form will include the following:

1. The nature and objectives, potential risks and benefits of the intended study.
2. The length of study and the likely follow-up required.
3. Alternatives to the proposed study. (This will include available standard and investigational therapies. In addition, patients will be offered an option of supportive care for therapeutic studies.)
4. The name of the investigator(s) responsible for the protocol.
5. The right of the participant to accept or refuse study interventions/interactions and to withdraw from participation at any time.

Before any protocol-specific procedures can be carried out, the consenting professional will fully explain the aspects of patient privacy concerning research specific information. In addition to signing the IRB Informed Consent, all patients must agree to the Research Authorization component of the informed consent form.

Each participant and consenting professional will sign the consent form. The participant must receive a copy of the signed informed consent form.

19.0 REFERENCES

1. Vivanco I, Sawyers CL: The phosphatidylinositol 3-Kinase-AKT pathway in human cancer. Nat Rev Cancer 2:489-501, 2002
2. Sawyers CL: Will Kinase Inhibitors Have a Dark Side? New England Journal of Medicine 355:313-315, 2006
3. Shaw RJ, Cantley LC: Ras, PI(3)K and mTOR signalling controls tumour cell growth. Nature 441:424-430, 2006
4. Engelman JA: Targeting PI3K signalling in cancer: opportunities, challenges, and limitations. Nat Rev Cancer 9:550-562, 2009
5. Samuels Y, Wang Z, Bardelli A, et al: High frequency of mutations of PIK3CA gene in human cancers. Science 304:554, 2004
6. Sansal I, Sellers WR: The biology and clinical relevance of the PTEN tumor suppressor pathway. J Clin Oncol 22:2954-2963, 2004
7. Bendell JC, Rodon J, Burris HA, et al: Phase I, dose-escalation study of BKM120, an oral pan-Class I PI3K inhibitor, in patients with advanced solid tumors. J Clin Oncol 30:282-90, 2012
8. Papadopoulos KP, Tabernero J, Markman B, et al: Phase I safety, pharmacokinetic, and pharmacodynamic study of SAR245409 (XL765), a novel, orally administered PI3K/mTOR inhibitor in patients with advanced solid tumors. Clin Cancer Res 20:2445-56, 2014
9. Shapiro GI, Rodon J, Bedell C, et al: Phase I safety, pharmacokinetic, and pharmacodynamic study of SAR245408 (XL147), an oral pan-class I PI3K inhibitor, in patients with advanced solid tumors. Clin Cancer Res 20:233-45, 2014
10. Hortobagyi GN, Piccart-Gebhart MJ, Rugo HS, et al: Correlation of molecular alterations with efficacy of everolimus in hormone receptor–positive, HER2-negative advanced breast cancer: Results from BOLERO-2. J Clin Oncol 31: abstr LBA509, 2013
11. Cohen Y, Shalmon B, Korach J, et al: AKT1 pleckstrin homology domain E17K activating mutation in endometrial carcinoma. Gynecol Oncol 116:88-91, 2010
12. Bleeker FE, Felicioni L, Buttitta F, et al: AKT1(E17K) in human solid tumours. Oncogene 27:5648-50, 2008
13. Do H, Solomon B, Mitchell PL, et al: Detection of the transforming AKT1 mutation E17K in non-small cell lung cancer by high resolution melting. BMC Res Notes 1:14, 2008
14. Malanga D, Scrima M, De Marco C, et al: Activating E17K mutation in the gene encoding the protein kinase AKT1 in a subset of squamous cell carcinoma of the lung. Cell Cycle 7:665-9, 2008
15. Carpten JD, Faber AL, Horn C, et al: A transforming mutation in the pleckstrin homology domain of AKT1 in cancer. Nature 448:439-444, 2007
16. O'Brien C, Wallin JJ, Sampath D, et al: Predictive Biomarkers of Sensitivity to the Phosphatidylinositol 3′ Kinase Inhibitor GDC-0941 in Breast Cancer Preclinical Models. Clinical Cancer Research 16:3670-3683, 2010
17. Fumagalli D, Gavin PG, Taniyama Y, et al: A rapid, sensitive, reproducible and cost-effective method for mutation profiling of colon cancer and metastatic lymph nodes. BMC Cancer 10:101, 2010
18. Kim MS, Jeong EG, Yoo NJ, et al: Mutational analysis of oncogenic AKT E17K mutation in common solid cancers and acute leukaemias. Br J Cancer 98:1533-5, 2008
19. Carpten JD, Faber AL, Horn C, et al: A transforming mutation in the pleckstrin homology domain of AKT1 in cancer. Nature 448:439-44, 2007
20. Hyman D, Smyth L, Bedard PL, et al: AZD5363, a catalytic pan-AKT inhibitor, in AKT1 E17K-mutation positive advanced solid tumors. Abstracts B109 and B181. AACR-NCI-EORTC Molecular Targets and Cancer Therapeutics Conference, Saturday Nov 7, 2015

21. Hyman DM, Solit DB, Arcila ME, et al: Precision medicine at Memorial Sloan Kettering Cancer Center: clinical next-generation sequencing enabling next-generation targeted therapy trials. Drug Discov Today 20:1422-8, 2015

22. Cheng DT, Mitchell TN, Zehir A, et al: Memorial Sloan Kettering-Integrated Mutation Profiling of Actionable Cancer Targets (MSK-IMPACT): A Hybridization Capture-Based Next-Generation Sequencing Clinical Assay for Solid Tumor Molecular Oncology. J Mol Diagn 17:251-64, 2015

23. Chang MT, Asthana S, Gao SP, et al: Identifying recurrent mutations in cancer reveals widespread lineage diversity and mutational specificity. 34:155-63, 2016

24. Lindsley CW: The Akt/PKB family of protein kinases: a review of small molecule inhibitors and progress towards target validation: a 2009 update. Curr Top Med Chem 10:458-77, 2010

25. Altomare DA, Testa JR: Perturbations of the AKT signaling pathway in human cancer. Oncogene 24:7455-64, 2005

26. Chang L, Chiang S-H, Saltiel AR: Insulin Signaling and the Regulation of Glucose Transport. Molecular Medicine 10:65-71, 2004

27. Marfella R, Rossi F, Giugliano D: Hyperglycemia and QT interval: time for re-evaluation. Diabetes Nutr Metab 14:63-5, 2001

28. van Noord C, Sturkenboom MC, Straus SM, et al: Serum glucose and insulin are associated with QTc and RR intervals in nondiabetic elderly. Eur J Endocrinol 162:241-8, 2010

29. Calo LA, Pessina AC: RhoA/Rho-kinase pathway: much more than just a modulation of vascular tone. Evidence from studies in humans. J Hypertens 25:259-64, 2007

30. Kempe DS, Siraskar G, Frohlich H, et al: Regulation of renal tubular glucose reabsorption by Akt2/PKBbeta. Am J Physiol Renal Physiol 298:F1113-7, 2010

31. Pal SK, Reckamp K, Yu H, et al: Akt inhibitors in clinical development for the treatment of cancer. Expert Opin Investig Drugs 19:1355-66, 2010

32. Cho DC, Hutson TE, Samlowski W, et al: Two phase 2 trials of the novel Akt inhibitor perifosine in patients with advanced renal cell carcinoma after progression on vascular endothelial growth factor-targeted therapy. Cancer 118:6055-62, 2012

33. Bosch A, Li Z, Bergamaschi A, et al: PI3K inhibition results in enhanced estrogen receptor function and dependence in hormone receptor-positive breast cancer. Sci Transl Med 7:283ra51, 2015

34. Ribas R, Pancholi S, Guest SK, et al: AKT Antagonist AZD5363 Influences Estrogen Receptor Function in Endocrine-Resistant Breast Cancer and Synergizes with Fulvestrant (ICI182780) In Vivo. Mol Cancer Ther 14:2035-48, 2015

35. Beer TM, Armstrong AJ, Rathkopf DE, et al: Enzalutamide in metastatic prostate cancer before chemotherapy. N Engl J Med 371:424-33, 2014

36. Carver BS, Chapinski C, Wongvipat J, et al: Reciprocal feedback regulation of PI3K and androgen receptor signaling in PTEN-deficient prostate cancer. Cancer Cell 19:575-86, 2011

20.0 APPENDICES

Appendix A Concomitant Treatment Cautions and Restrictions for AZD5363

Appendix B RECIST v1.1

Appendix C PCWG3

Appendix D RANO

Appendix E Categorization of Quantitative Protein Urine Analysis

Appendix F Patient instructions for the management of Diarrhea
