# Supplementary material for: AKT mutant allele-specific activation dictates pharmacologic sensitivities
Source: Nat Commun. 2022 Apr 19;13:2111. doi: 10.1038/s41467-022-29638-1 (PMC9018718; doi:10.1038/s41467-022-29638-1)
Supplement: Supplementary file 3 — Reporting Summary [file 41467_2022_29638_MOESM3_ESM.pdf]

nature research

Corresponding author(s): Barry S. Taylor

Last updated by author(s): Jan 24, 2022

Reporting Summary

Nature Research wishes to improve the reproducibility of the work that we publish. This form provides structure for consistency and transparency in reporting. For further information on Nature Research policies, see our Editorial Policies and the Editorial Policy Checklist.

Statistics

For all statistical analyses, confirm that the following items are present in the figure legend, table legend, main text, or Methods section.

n/a	Confirmed	
☐	☒	The exact sample size (n) for each experimental group/condition, given as a discrete number and unit of measurement
☐	☒	A statement on whether measurements were taken from distinct samples or whether the same sample was measured repeatedly
☐	☒	The statistical test(s) used AND whether they are one- or two-sided<br>*Only common tests should be described solely by name; describe more complex techniques in the Methods section.*
☐	☒	A description of all covariates tested
☐	☒	A description of any assumptions or corrections, such as tests of normality and adjustment for multiple comparisons
☐	☒	A full description of the statistical parameters including central tendency (e.g. means) or other basic estimates (e.g. regression coefficient) AND variation (e.g. standard deviation) or associated estimates of uncertainty (e.g. confidence intervals)
☐	☒	For null hypothesis testing, the test statistic (e.g. F, t, r) with confidence intervals, effect sizes, degrees of freedom and P value noted<br>*Give P values as exact values whenever suitable.*
☒	☐	For Bayesian analysis, information on the choice of priors and Markov chain Monte Carlo settings
☒	☐	For hierarchical and complex designs, identification of the appropriate level for tests and full reporting of outcomes
☒	☐	Estimates of effect sizes (e.g. Cohen's d, Pearson's r), indicating how they were calculated

Our web collection on statistics for biologists contains articles on many of the points above.

Software and code

Policy information about availability of computer code

Data collection	No code used for data collection.
Data analysis	UCSF Chimera (version 1.12), Modeller (version 1.19), GROMACS (version 5.1.4), SSPIDER (version 2), GraphPad PRISM (version 8), RECIST v1.1., TM-align software (version 20180816), I-TASSER (version 5.1), R (version 3.4.1), additional R packages: data.table (v1.14.0), ggplot2 (v3.3.3), cowplot (v1.1.1), Rpdb (v2.3), ggrepel (v0.9.1), ggsign if (v0.6.0), here (v1.0.1). Source code for these analyses is available at https://github.com/agorelick/akt1.

For manuscripts utilizing custom algorithms or software that are central to the research but not yet described in published literature, software must be made available to editors and reviewers. We strongly encourage code deposition in a community repository (e.g. GitHub). See the Nature Research guidelines for submitting code & software for further information.

Data

Policy information about availability of data

All manuscripts must include a data availability statement. This statement should provide the following information, where applicable:

- Accession codes, unique identifiers, or web links for publicly available datasets
- A list of figures that have associated raw data
- A description of any restrictions on data availability

Source data are provided with this paper and are available here: https://doi.org/10.5281/zenodo.5111040. All mutational data from the prospective sequencing cohort is available for download at http://cbioportal.org/. All other genomic and clinical data are available from public sources (MC3; https://gdc.cancer.gov/about-data/publications/mc3-2017) or accompanies the manuscript and is available as Supplementary information, including the clinical trial protocol. Other materials, code, and deidentified patient-level clinical data not otherwise presented in the Supplementary information is available upon request.

Field-specific reporting

Please select the one below that is the best fit for your research. If you are not sure, read the appropriate sections before making your selection.

- [x] Life sciences
- [ ] Behavioural & social sciences
- [ ] Ecological, evolutionary & environmental sciences

For a reference copy of the document with all sections, see nature.com/documents/nr-reporting-summary-flat.pdf

Life sciences study design

All studies must disclose on these points even when the disclosure is negative.

Sample size	Sample size was not pre-determined; all available specimens were utilized.
Data exclusions	No exclusion criteria were specified for the study population.
Replication	Reproducibility of data was ascertained by performing experiments at least three times using different methods, reagents and at different data points, as also described in the Methods section.
Randomization	No randomization of data was performed.
Blinding	No blinding of data was performed.

Reporting for specific materials, systems and methods

We require information from authors about some types of materials, experimental systems and methods used in many studies. Here, indicate whether each material, system or method listed is relevant to your study. If you are not sure if a list item applies to your research, read the appropriate section before selecting a response.

Materials & experimental systems

n/a	Involved in the study	
☐	☒	Antibodies
☐	☒	Eukaryotic cell lines
☒	☐	Palaeontology and archaeology
☒	☐	Animals and other organisms
☐	☒	Human research participants
☐	☒	Clinical data
☒	☐	Dual use research of concern

Methods

n/a	Involved in the study	
☒	☐	ChIP-seq
☒	☐	Flow cytometry
☒	☐	MRI-based neuroimaging

Antibodies

Antibodies used

The antibodies are described under Methods section, and have been validated by the companies in-house as well as in the publications cited in the product literature.
1. pAkt (T308) monoclonal antibody (D25E6), Cell Signaling Technology (CST) catalog # 13038, lot # 5.
2. pAkt (S473) polyclonal antibody (D9E), Cell Signaling Technology catalog # 4060, lot # 23.
3. pGSK3-beta (S9) polyclonal antibody, Cell Signaling Technology catalog # 9336, lot # 12.
4. p-PRAS40 (T246) polyclonal antibody, Cell Signaling Technology catalog # 2640, lot # 5.
5. pS6RP (S240/244) polyclonal antibody, Cell Signaling Technology catalog # 2215, lot # 18.
6. GSK3-beta monoclonal antibody (27C10), Cell Signaling Technology catalog # 9315, lot # 14.
7. PRAS40 polyclonal antibody, Cell Signaling Technology catalog # 2610, lot # 2.
8. S6-RP monoclonal antibody (5G10), Cell Signaling Technology catalog # 2217, lot # 7.
9. V5 probe monoclonal antibody (E10), Santa Cruz Biotechnology catalog # sc-81594, lot # E1816.
10. Beta-actin monoclonal antibody (C4), Santa Cruz Biotechnology. Catalog # sc-47778, lot # J1916.
11. Goat anti-mouse IgG secondary antibody, Alexa Fluor 488. ThermoFisher Scientific. Catalog # A11001. Lot # 1834337.
12. Goat anti-rabbit IgG secondary antibody Alexa Fluor 594. ThermoFisher Scientific. Catalog # R37117. Lot # 1856877.

Validation

1. pAkt (T308) monoclonal antibody (D25E6) validated by western blot analysis of extracts from NIH3T3 cells (untreated or treated with PDGF); and untreated LNCAP and PC-3 cells. This antibody was validated for immunofluorescence by confocal immunofluorescent analysis of C2C12 cells treated with either Insulin or LY294002.
2. pAkt (S473) polyclonal antibody (D9E), validated by western blot analysis of extracts from PC-3 cell, untreated or LY294002/wortmannin-treated; and NIH3T3 cells, serum-starved or PDGF-treated.
3. pGSK3-beta (S9) polyclonal antibody, validated by western blot analysis of extracts from NIH3T3 cells either untreated or treated

	with lambda-phosphatase.<br>4. p-PRAS40 (T246) polyclonal antibody, validated by western blot analysis of extracts from NIH3T3 cells, serum-starved, and then either left untreated or treated with Insulin. This antibody was additionally validated for specificity using a phospho-PRAS40-specific blocking peptide.<br>5. pS6RP (S240/244) polyclonal antibody, validated by western blot analysis of extracts from 293 cells, either untreated or treated with 20% FBS for various time periods.<br>6. GSK3-beta monoclonal antibody (27C10), validated by western blot analysis of extracts from HeLa, NIH3T3, COS, C6 and 293 cells.<br>7. PRAS40 polyclonal antibody, validated by western blot analysis of extracts from various cell types.<br>8. S6-RP monoclonal antibody (5G10), validated by western blot analysis of extracts from HeLa, NIH3T3, PC12 and COS cells.<br>9. V5 probe monoclonal antibody (E10), validated by western blot analysis of extracts from 293T cells that were either non-transfected, or transfected with V5-tagged human karyopherin alpha6.<br>10. Beta-actin monoclonal antibody (C4), validated by western blot analysis of whole cell extracts from HeLa, Sol8, C32, NIH3T3 and KNRK cells.

Eukaryotic cell lines

Policy information about cell lines

Cell line source(s)	a) 293 FT cells were from ATCC.<br>b) MCF10a cells were from David Solit laboratory<br>c) BaF3's were from Ross Levine's lab.
Authentication	All cell lines have been authenticated by short tandem repeat analysis.
Mycoplasma contamination	All cell lines were confirmed tested negative for mycoplasma contamination.
Commonly misidentified lines<br>(See ICLAC register)	No commonly mis-identified cell lines were utilized.

Human research participants

Policy information about studies involving human research participants

Population characteristics	Age at the time of prospective sequencing: Mean of 62 years (standard deviation 15 years).<br>Male/female ratio: 45.2%/54.8%.<br>Additional details available for trial patients in Supplementary Table 3.
Recruitment	In addition to publicly available genomic data, a subset of patient underwent prospective sequencing as part of their clinical care at Memorial Sloan Kettering Cancer Center (MSKCC) from January 2014 to May 2018; and from October 2017 to April 2020 for the investigator-initiated trial. All such patients whose tumor sequencing was performed with a matched normal sample were included. In essence, eligible patients 18 years of age or older were recruited on the basis of a pathologically confirmed diagnosis of a recurrent or metastatic advanced solid tumor with a somatic mutation in AKT1, AKT2, or AKT3 detected by prospective clinical sequencing.<br>[Outline any self-selection bias or other biases that may be present and how these are likely to impact results].
Ethics oversight	MSKCC Institutional Review Board

Note that full information on the approval of the study protocol must also be provided in the manuscript.

Clinical data

Policy information about clinical studies

All manuscripts should comply with the ICMJEguidelines for publication of clinical research and a completedCONSORT checklist must be included with all submissions.

Clinical trial registration	NCT01775072,<br>NCT03310541
Study protocol	Included as a "manuscript related file" and available at clinicaltrials.gov, NCT03310541.
Data collection	Locale of data collection: Memorial Sloan Kettering Cancer Center.<br>Dates of recruitment for prospectively characterized patients utilized here were from January 2014 to May 2018, and for the investigator-initiated trial, from October 2017 to April 2020.
Outcomes	The primary endpoint was objective response rate (ORR) defined as the proportion of patients with a confirmed complete response (CR) or partial response (PR) using either modified RECIST v1.1 or ≥ 50% PSA decrease from baseline (in prostate cancer patients without visceral and/or nodal disease at baseline). Secondary endpoints included assessment of toxicity according to NCI common toxicity criteria (CTC) version 4.0, progression-free survival (PFS), and clinical benefit rate (CBR) defined as complete response, partial response, or stable disease at 24 weeks
